# Reaction of the endogenous regulatory mechanisms to early weekday wakeups: a review of its popular explanations in light of model-based simulations

**DOI:** 10.3389/fnetp.2023.1285658

**Published:** 2023-12-15

**Authors:** Arcady A. Putilov

**Affiliations:** ^1^ Laboratory of Nanobiotechnology and Biophysics, North-Caucasus Federal University, Stavropol, Russia; ^2^ Laboratory of Sleep/Wake Neurobiology, Institute of Higher Nervous Activity and Neurophysiology of the Russian Academy of Sciences, Moscow, Russia

**Keywords:** model, simulation, circadian clocks, sleep–wake regulation, sleep deficiency

## Abstract

**Introduction:** Several widely held explanations of the mechanisms underlying the responses of endogenous sleep–wake-regulating processes to early weekday wakeups have been proposed. Here, they were briefly reviewed and validated against simulations based on the rhythmostatic version of a two-process model of sleep–wake regulation.

**Methods:** Simulated sleep times on weekdays and weekends were compared with the times averaged over 1,048 samples with either earlier or later weekday risetimes. In total, 74 paired samples were collected before and during lockdown, and 93 paired samples were collected during early and later school start times.

**Results:** The counterintuitive predictions of the simulations included the following: 1) only one night of *ad lib* sleep is sufficient to restore the endogenously determined sleep times after 1 day/5 days of larger/smaller reduction/extension of the sleep/wake phase of the circadian sleep–wake cycle; 2) sleep loss on weekdays is irrecoverable; 3) irrespective of the amount of such deadweight loss, sleep on weekends is not prolonged; and 4) the control of the circadian clocks over the sleep–wake cyclicity is not disrupted throughout the week.

**Discussion:** The following popular explanations of the gaps between weekends and weekdays in sleep timing and duration were not supported by these simulations: 1) early weekday wakeups cause “social jetlag,” viewed as the weekend and weekday (back and forth) shifts of the sleep phase relative to the unchanged phase of the circadian clocks, and 2) early weekday wakeups cause an accumulation of “sleep debt paid back” on weekends, or, in other terms, people can “catch-up” or “compensate” sleep on weekends.

## 1 Introduction

The two-process model of sleep–wake regulation developed in 1984 by Borbély et al. (Daan et al., 1984) exemplifies a successful story of mathematical modeling and model-based simulations in the field of biological clocks and sleep research. For 4 decades, this model has been the major contributor to our understanding of the mechanism of regulation of the sleep–wake cycle. Unfortunately, the model-based simulations were rarely applied in questionnaire studies of human sleep timing and duration on weekdays and weekends.

Several concepts were recently introduced to explain the mechanisms underlying the responses of the endogenous sleep–wake regulators to early wakeups on weekdays, followed by *ad lib* sleep on weekends. They can be grouped into concepts explaining the responses of sleep–wake regulation to the conflicts between social and internal (biological) timing and concepts of recovery weekend sleep. The concepts of the first group became widely known under the term “social jetlag.” They postulate the stability of the phase of the circadian clocks and, in contrast, the back and forth shifts of the phase of the sleep–wake cycle relative to this stable clock phase (twice a week, on weekends and weekdays, respectively) (Wittmann et al., 2006). The second group of concepts explicitly or implicitly refer to the two-process model because they postulate a hypothetical accumulation of “sleep debt” during weekdays with its following “paying off” during the weekend (Roenneberg et al., 2012; Kitamura et al., 2016; Shen et al., 2021). Such weekend sleep–wake behavior is also often termed “catch-up” (Kim et al., 2011) or “compensatory” sleep (Åkerstedt et al., 2019), usually without any mention about the preceding accumulation of “sleep debt.”

The authors of the two-process model noticed a close resemblance between their model and a thermostat. They called it “somnostat,” because their model predicts the so-called hysteresis when “the system acts like a thermostat that switches off at a higher threshold than it switches on” (Daan et al., 1984). As early as in 1971, Hubertus Strughold (“the father of space medicine”) introduced the term “rhythmostasis” to define the capacity of the body to keep its rhythms nearly constant (Strughold, 1971). Such a basic feature of the regulation of biological oscillations was accounted for in one of the versions of the two-process model by postulating that the parameters of such a “somnostat” (11) (Daan et al., 1984) are modulated by circadian clocks (12) (Putilov, 1995).

The present paper was purposed on the validation of the concepts explaining the gaps between weekends and weekdays in sleep times against simulations of these times with the rhythmostatic model (11, 12) (Putilov, 1995). The datasets collected from two recent publications reporting results of such simulations (Putilov, 2022; Putilov, 2023) were enlarged and reanalyzed to compare the similarity of sleep times from these datasets with the sleep times predicted by the model. The concepts explaining the gaps between weekends and weekdays in sleep times were discussed in light of the simulation results, and several counterintuitive but testable predictions of the rhythmostatic model were highlighted to pave the way for experimental studies aimed at providing empirical support.

## 2 Results

This section addresses the question of whether calculations and simulations based on the two-process model (11, 12) can provide support for the most popular explanations of the gaps between sleep times on weekdays and weekends. The results include the results of model-based computations of sleep times (*in silico* study) and the results of model-based simulations of sleep times from an empirical dataset (simulation study). The model parameters for the present computations and simulations are given in Table 1. In the classical version of the two-process model (Daan et al., 1984), the process of sleep–wake regulation, *S(t)*, was represented by an inverse exponential buildup during the wake phase (11a) and exponential decay during the sleep phase (11b). Since it is plausible to expect that the parameters of this process of sleep–wake regulation are additionally modulated by body clocks, such a modulation is included in the form of a 24-h sine-form function, *C(t)* (12) (Putilov, 1995). At baseline, the process *S(t)* alternates between *S*_*d*_ *(t)* and *S*_*b*_ *(t),* which are the highest expected buildup and the lowest expected decay of *S(t)*, i.e., sleep onset and offset, respectively. These parameters are determined by the endogenous sleep–wake-regulating mechanism named “rhythmostat” (Putilov, 1995).

**TABLE 1:** Parameters of the rhythmostat model used for computations and simulations.

Parameters		Initial (PoW and EWU)			Three ERTs		
Inverse exponential buildup (1a) and exponential decay phases (1b) of *S(t)*	*S*_*b*_ (lowest allowed decay), rSWA	0.75	0.75	0.75	0.75	0.75	0.75
	*S*_*d*_ (highest allowed buildup), rSWA	2.50	2.50	2.50	2.50	2.50	2.50
	*S*_*l*_ (lower asymptote), rSWA	0.70	0.70	0.70	0.70	0.70	0.70
	*S*_*u*_ (upper asymptote), rSWA	4.50	4.50	4.50	4.51	4.51	4.51
	*T*_*d*_ (phase constant for decay), h	1.95	1.95	1.95	2.30	2.30	2.30
	*T*_*b*_ (phase constant for buildup), h	27.04	27.04	27.04	24.75	24.75	24.75
24-h sine waveform modulation 2) of parameters of buildup (1a) and decay (1b)	φ_max_ (circadian peak), clock h	15.00	15.00	15.00	16.00	16.00	16.00
	*A* (circadian amplitude), rSWA	0.50	0.50	0.50	0.50	0.50	0.50
	*τ* (entrained circadian period), h	24.00	24.00	24.00	24.00	24.00	24.00
	*k* (circadian term, two-fold impact)	2.00	2.00	2.00	2.00	2.00	2.00
Initial times for buildup (1a) and decay (1b)	*t2*, clock h	23.00	23.00	23.00	24.00	24.00	24.00
	*t1*, clock h	7.00	7.00	7.00	9.00	9.00	9.00
Prolongation of wakefulness (PoW = +), h		+1.00	0.00	+1.00	0.00	0.00	0.00
Earlier wakeups/earlier risetimes (EWU = −/ERT = −), clock h		7.00	6.00	6.00	6.00	7.00	8.00
Advance of EWU/ERT relative to *t1* or *t2*, h		0.00	−1.00	−1.00	−1.00	−2.00	−3.00

Initial (PoW and EWU): Parameters of the model of the sleep–wake-regulating process *S(t)* were derived by Putilov (1995) by simulating data on sleep durations after prolongation of wakefulness (PoW) and on relative slow-wave activity (rSWA) in naps and two extended sleep episodes (mean rSWA = 1 in a baseline night sleep episode). These initial parameters were used to calculate the responses to PoW = +1.00 h, earlier wakeup (EWU = −1.00 h), and both PoW = +1.00 h and EWU = −1.00 h. In all these calculations, *t2* and *t1* for the baseline conditions were proposed to be identical (23.00 and 7.00, respectively). The difference was limited to differences in the extension of duration of the wake phase of the sleep–wake cycle relative to *t2* = 23.00 and/or in the reduction in the duration of the sleep phase relative to *t1* = 7.00 (PoW = +1.00 h and PoW = 0.00 h, and/or EWU = −1.00 h and EWU, 0.00 h, respectively). Three ERTs: The initial parameters of the model (Putilov, 1995) were slightly modified for calculating the responses to different earlier risetimes (ERTs) by taking into account the difference between the experimental sleep durations simulated by Putilov (1995) and times in bed in studies reporting only bedtimes and risetimes (8.00 h and 9.00 h, respectively). The difference was limited to differences in the extent of the reduction in the duration of sleep phase relative to baseline *t1* = 9.00 by advancing risetime on 5 weekdays (either ERT = −3.00 h or −2.00 h or −1.00 h). Clock time is given in decimal hours.

### 2.1 *In silico* study of the effects of manipulations with bedtimes and risetimes

People do not always obey the signal of falling asleep sent by this internal device. Instead, they regularly find themselves in a situation when they are forced to (or eager to voluntarily) extend the duration of the wake phase by delaying the time to go to bed and/or reduce the duration of the sleep phase by advancing the time to get up. For instance, almost everyone practiced such kinds of get-ups advancing on five work/school days and sleeping *ad lib* only during the following 2-day weekend.

#### 2.1.1 Effects of change in sleep phase durations: computations vs. explanations

**FIGURE 1:**
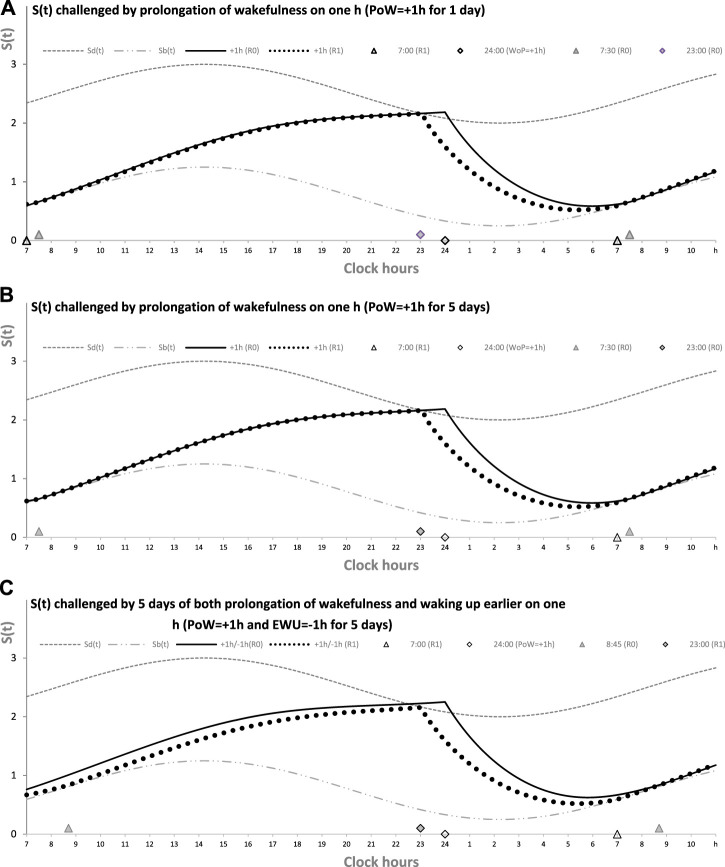
Sleep–wake cycles challenged by 1 or 5 days with the prolongation of wakefulness. Two phases of the process of sleep–wake regulation *S(t)* can be simply described as daily alternations of an inverse exponential buildup during the wake phase (11a) and an exponential decay during the sleep phase (11b), but only in the absence of the circadian clocks. During their presence in the body, the parameters of *S(t)* are proposed to be additionally modulated by these clocks. A sine-form function with a 24-h period, *C(t)* (12), accounts for this modulation. *S*_*d*_ *(t)* and *S*_*b*_ *(t)*: The highest allowed buildup and the lowest allowed decay of *S(t)*, respectively, i.e., sleep onset and offset, respectively, determined by this endogenous sleep–wake-regulating process (1, 2) (Putilov, 1995). PoW: Forced or voluntary prolongation of wakefulness beyond the highest allowed buildup, i.e., further increase in *S(t)* after crossing *S*_*d*_ *(t)*. Two sleep–wake cycles (R0 and R1) illustrate that the durations of reestablishment of normal (endogenously determined) sleep times after only 1 day (A) and 5 (week)days of PoW = +1.00 h B). The rate of this reestablishment is practically identical due to the permanent circadian modulation, *C(t)*, of the parameters of *S(t)*. Namely, it takes only 1 day with *ad lib* sleep to restore the baseline times of sleep onset, offset, and duration (23:00, 7:00, and 8.00 h, respectively), i.e., the alternations of two phases—sleep and wake—of this process between *S*_*d*_ *(t)* and *S*_*b*_ *(t)*. It should be noted that PoW for either 1 or 5 days (A or B) leads to further buildup of *S(t)* that can be interpreted as accumulation of “sleep debt” that is “paid off” during the following (recovery *ad lib*) sleep. This sleep is predicted to be longer than the sleep started at the same time point at *S*_*d*_ *(t)*. In these computations, any sleep episode is terminated at *S*_*b*_ *(t)* (C). The processes of recovery of normal (endogenously determined) sleep duration and timing are also practically identical after 5 (week)days of combination of PoW = +1.00 h with earlier wakeups (EWU = −1.00 h). Again, due to the circadian modulation *C(t)* of the parameters of *S(t)*, it takes only 1 day with *ad lib* sleep to restore the baseline times of sleep onset, offset, and duration (23:00, 7:00, and 8.00 h, respectively) determined by the endogenous sleep–wake regulator (Putilov, 1995). However, the preceding *ad lib* sleep reflects the extent of shortening of the sleep phase. It is terminated either at 7:30 in **(A,B)** or later, at 8:45, in **(C)**. See also the initial model parameters for these calculations in Table 1.

Figure 1A illustrates this case of prolongation of wakefulness (PoW) beyond the highest expected buildup, i.e., further increase in *S(t)* after crossing *S*_*d*_ *(t)*. Such an additional buildup of *S(t)* can be interpreted as the accumulation of “sleep debt.” Supplementary Figure SA1 illustrates that it is “paid off” during the following (recovery) sleep that might be longer than the sleep started after reaching *S*_*d*_ *(t)*. However, such recovery sleep is similar to the baseline (unchallenged) sleep in the mechanism that terminates sleep. The rhythmostat terminates any *ad lib* sleep at *S*_*b*_ *(t)*. The two sleep–wake cycles shown in Figures 1A, B illustrate that due to the circadian modulation *C(t)* of the parameters of *S(t)*, it takes only one night of *ad lib* sleep to return to the baseline sleep times, i.e., sleep onset, offset, and duration at 23:00, 7:00, and 8.00 h, respectively. The normal, endogenously determined alternation of two phases of this process is fully restored because the process starts its decay at *S*_*d*_ *(t)* to be switched to its buildup at *S*_*b*_ *(t)*.

Figure 1 illustrates that in any of three computations (1A, 1B, and 1C), such a process of restoration of the baseline (endogenously determined) duration and timing of sleep is identical, i.e., after PoW for only 1 day (Figure 1A), after 5 days of PoW (Figure 1B), and even after combining this PoW with earlier waking-up (EWU) for 5 days (Figure 1C). Two sleep-wake cycles illustrate that due to the circadian modulation *C(t)* of the parameters of *S(t)*, it takes only one night of *ad lib* sleep to return to the baseline times of sleep onset, offset, and duration (23:00, 7:00, and 8.00 h, respectively).

**FIGURE 2:**
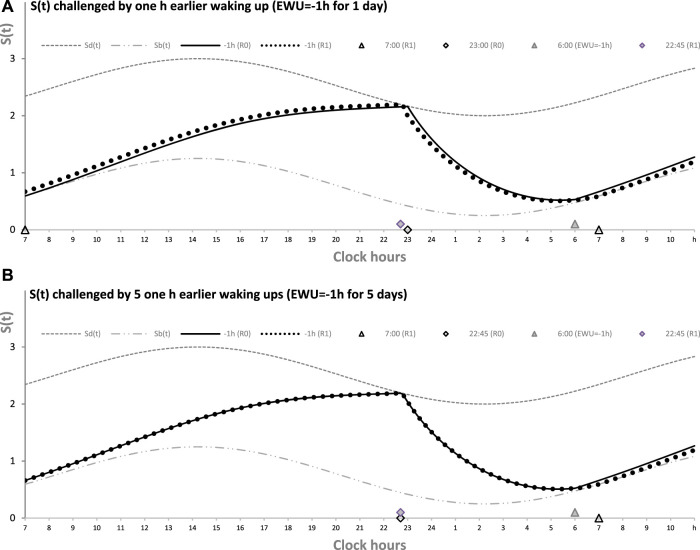
Sleep–wake cycles challenged by 1 or 5 days with earlier weekday wakeups. The reestablishment of normal (endogenously determined) sleep timing and duration after earlier wakeup (EWU = –1 h) either for only 1 day **(A)** or for 5 (week)days in a row **(B)**. Two sleep–wake cycles (R0 and R1) illustrate that due to the circadian modulation *C(t)* of the parameters of *S(t)*, it takes only 1 day with *ad lib* sleep to restore the baseline times of sleep onset, offset, and duration (23:00, 7:00, and 8.00 h, respectively), i.e., the alternations of two phases—sleep and wake—of the sleep–wake cycle between *S*_*d*_ *(t)* and *S*_*b*_ *(t)* determined by the endogenous sleep–wake regulator (Putilov, 1995). Sleep is always started at *S*_*d*_ *(t)* to be terminated either by EWU or by this endogenous sleep–wake-regulator at *S*_*b*_ *(t)* in the case of *ad lib* sleep. Such a sleep is started at 22:45 on any of the days with EWU at 6:00. It should be noted that since PoW = 0 h before and after EWU (i.e., sleep onset is set at 23:00 at baseline and remains to be terminated even earlier, at *S*_*d*_ *(t)*, after EMU), no such EWU can lead to the accumulation of “sleep debt.” Therefore, there is nothing to be “paid off” during the following *ad lib* sleep. Such a sleep can be named “relaxatory” rather than “recovery” sleep. See also the legend of Figure 1 and the initial model parameters used for these calculations in Table 1.

Figure 2 illustrates the process of restoration of the baseline (endogenously determined) duration and timing of sleep after EWU for 1 and 5 days (Figures 1A, B, respectively). It has to be noted that since PoW = 0 h in these cases (i.e., sleep onset is set at 23:00 h), such EWU cannot lead to the accumulation of “sleep debt,” and, consequently, there is nothing to be “paid off” during the following *ad lib* sleep.

To sum up, these computations of the effects of voluntary (forced) manipulation with the times to go to bed and/or getting up showed that, after any such manipulation, it takes only one night of *ad lib* sleep to return to the baseline (endogenously determined) times of sleep onset, offset, and duration. Moreover, the sleep–wake cycle always remains in sync with the circadian clocks. Their synchrony cannot be disturbed by the voluntary (forced) shifts of the times to go to bed at later hours and the shifts of the times to get up at earlier hours. The time to get up and the time to go to bed are set by the rhythmostat because the circadian clocks do not stop their modulating influence on the sleep–wake process. If voluntary PoW or EWU occurs to replace sleep, the clocks modulate the time course of the regulated process during the wake phase of the cycle to restore normal sleep and wake timing. Consequently, the delaying or advancing shifts of sleep onset and offset after PoW and EWU, respectively, cannot be interpreted as the shift in the phase of the sleep–wake regulating process, *S(t)*, relative to the phase of the body clocks represented by *C(t)*. The parameters of *S(t)* are always controlled by the circadian clocks in such a way that only one night of *ad lib* sleep is required to restore baseline times of sleep onset, offset, and duration after any voluntary (forced) extension/reduction of the wake/sleep phase of the sleep–wake cycle.

#### 2.1.2 Computations of the sleep–wake cycles differed in weekday risetimes

**FIGURE 3:**
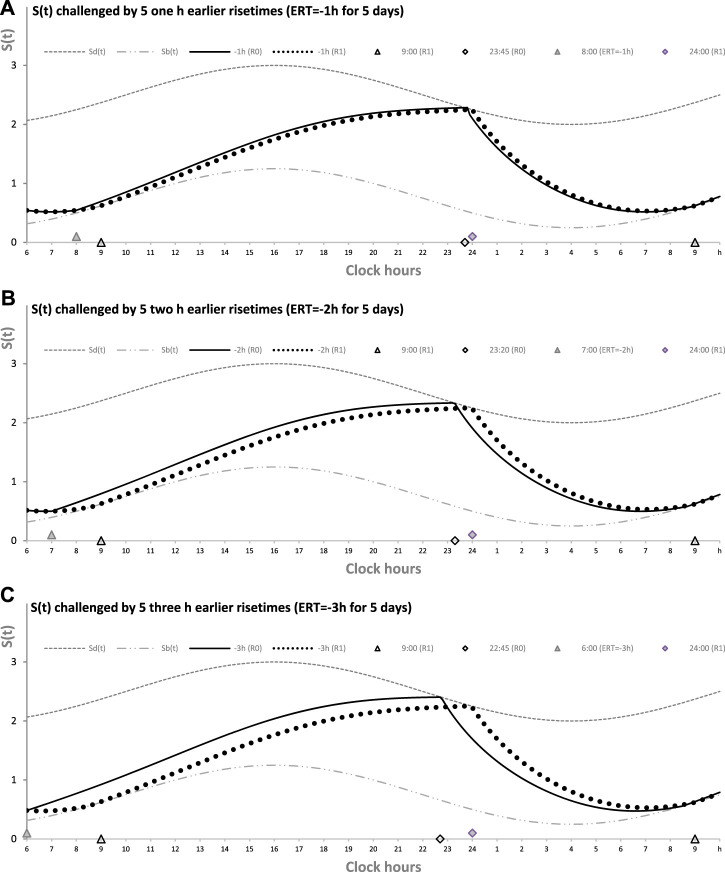
Sleep–wake cycles challenged by different earlier weekday risetimes. R0 and R1: Two sleep–wake cycles after challenging the cycle by 5 days with earlier weekday risetimes (ERT). *S(t)*: Two-phase process of sleep–wake regulation, i.e., an inverse exponential buildup (11a) and exponential decay (11b), i.e., the wake and sleep phases of the sleep–wake cycle, respectively. The parameters of this process are modulated by a sine-form function with a 24-h period, *C(t)* (12). *S*_*d*_ *(t)* and *S*_*b*_ *(t)*: The highest allowed buildup and the lowest allowed decay of *S(t)*, respectively, i.e., bedtime and risetime, BT and RT, respectively, determined by the endogenous sleep–wake regulator (Putilov, 1995). In three calculations **(A–C)**, ERTs are suggested to be either large or usual or small; i.e., RT is advanced by 3 h or 2 h or 1 h, respectively, relative to RT on vacation days with *ad lib* BT and RT. Due to the influence of the sleep–wake-regulating mechanism, sleep is initiated at *S*_*d*_ *(t)* on any of the 5 days of the week, but it is terminated either by EWU or at *S*_*b*_ *(t)* after *ad lib* sleep. Therefore, this sleep is started approximately at 22:45, 23:20, and 23:45 on the last day with EWU at 6:00, 7:00, and 8:00, respectively. Due to the circadian modulation *C(t)* of the parameters of *S(t)*, it takes only 1 day with *ad lib* sleep to restore the baseline bedtimes and risetimes and time in bed (24:00, 9:00, and 9.00 h, respectively), i.e., the alternation of two phases—sleep and wake—of the sleep–wake cycle between *S*_*d*_ *(t)* and *S*_*b*_ *(t)* determined by the endogenous sleep–wake regulator (Putilov, 1995). It should be noted that no EWU can lead to the accumulation of “sleep debt” because PoW = 0 h on any of the days before and after EWU; i.e., sleep onset is set at 23:00 at the baseline and remains to be terminated at *S*_*d*_ *(t)* after EWU. Therefore, since there is nothing to be “paid off” during *ad lib* sleep after days with EWU without PoW, such a sleep can be named “relaxatory” rather than “recovery” sleep. See also the legends to Figures 1, 2 for other notes, Table 1 for the model parameters, and Table 2 for sleep times for 10 days.

**FIGURE 4:**
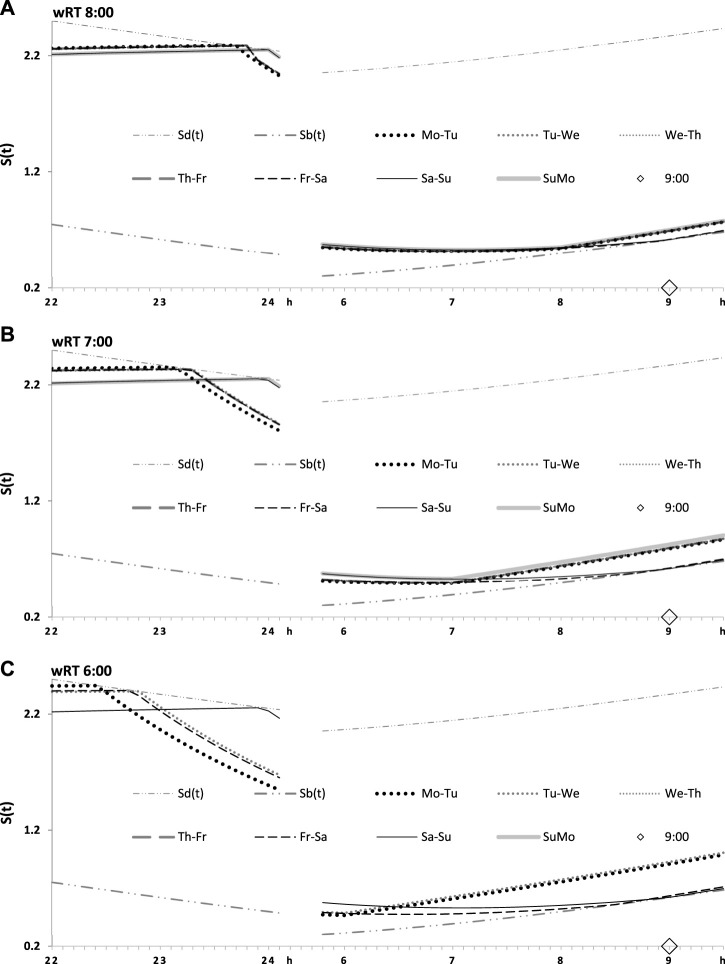
Seven simulated sleep–wake cycles on two subintervals (around bedtimes and risetimes). Simulations from Figure 3 are shown for 7 days of the week on two short time intervals, from 22:00 to 24:00 (around bedtime) and from 6:00 to 9:30 (around risetime the following day). **(A–C)** Simulations for weekday risetimes (wRT) at 8:00, 7:00, and 6:00 respectively. See also Figure 4 and Table 2.

**FIGURE 5:**
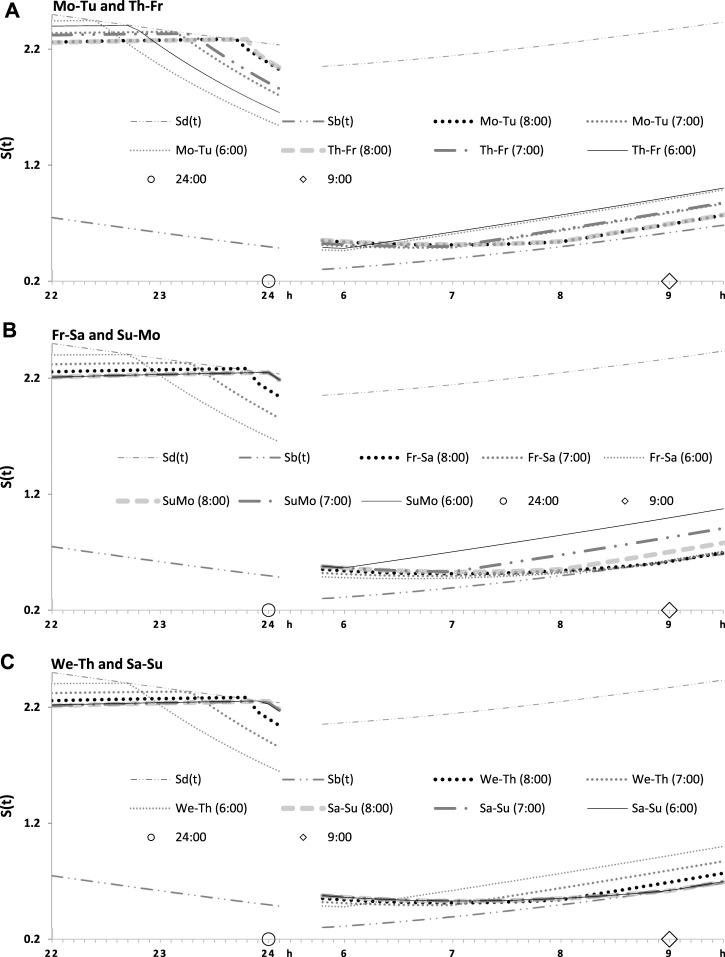
Two simulated sleep–wake cycles on two subintervals (around bedtimes and risetimes). Simulations from Figures 3, 4 are shown for 2 days of the week on two short time intervals, from 22:00 to 24:00 (around bedtime) and from 6:00 to 9:30 (around risetime at the following day). **(A–C)** Two weekdays, two transitions between weekdays and weekends, and one weekday and one weekend, respectively, in the simulations of three different wRTs, 8:00, 7:00, and 6:00.

**FIGURE 6:**
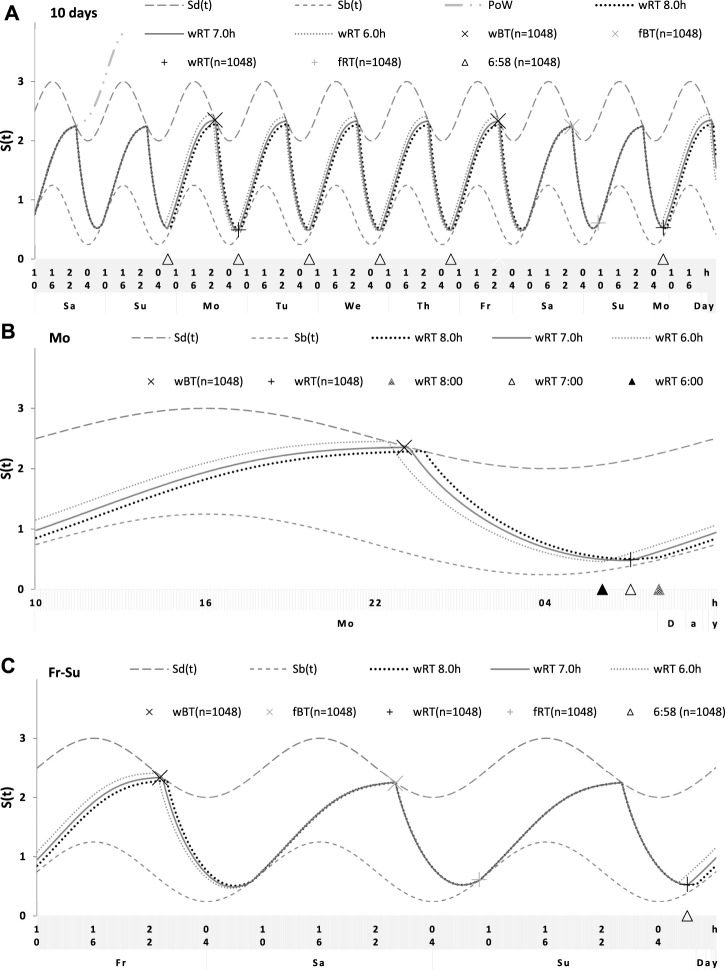
Simulations of the sleep–wake cycles in comparison of sleep times from the whole dataset. **(A)** Simulations of the sequence of 10 sleep–wake cycles consisting of 2 last days of vacation (Sa and Su), 5 weekdays (Mo–Fr), two weekend days (Sa–Su), and the first weekday of the next week (Mo). **(B, C)** Two subintervals of this 10-day interval, for a weekday **(B)** and the last 3 days **(C)**. *S(t)*: Two-phase process of sleep–wake regulation, i.e., an inverse exponential buildup (11a) and exponential decay (11b) in which parameters are modulated by a sine-form function with 24-h period (12). *S*_*d*_ *(t)* and *S*_*b*_ *(t)*: The highest expected buildup and decay of *S(t)* (i.e., bedtime and risetime, BT and RT, respectively, determined by the sleep–wake regulator). PoW: An example of buildup caused by the prolongation of wakefulness beyond the highest expected buildup, i.e., further buildup of *S(t)* at an approximately 1-day interval; wBT and fBT and wRT and fRT: Weekday and weekend bedtimes and risetimes, respectively, in the whole dataset (n = 1,048). For the model parameters, sleep times for each of 10 simulated cycles, and this whole dataset, see also Tables 1–3.

**FIGURE 7:**
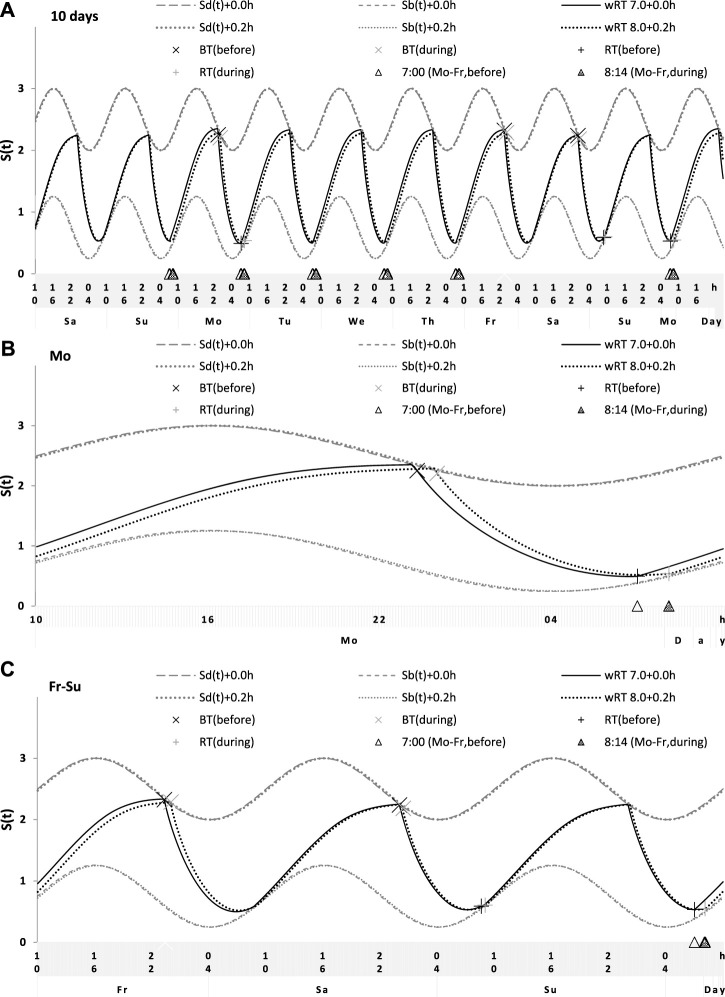
Simulation of the sleep–wake cycles observed before and during lockdown. See Table 4 for the comparison of sleep times before and during lockdown (74 paired samples). **(A)** Simulations of the sequence of 10 sleep–wake cycles consisting of 2 last days of vacation (Sa and Su), 5 weekdays (Mo–Fr), two weekend days (Sa–Su), and the first weekday of the next week (Mo). **(B, C)** Two subintervals of this 10-day interval, for a weekday **(B)** and the last 3 days **(C)**.

**FIGURE 8:**
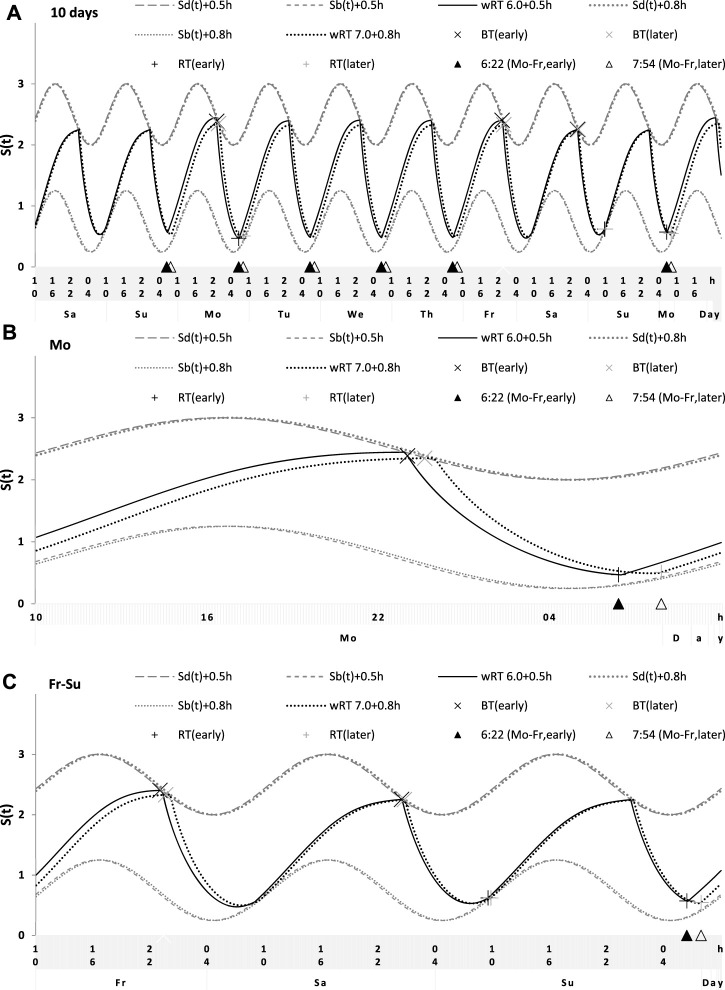
Simulation of the sleep–wake cycles during early and later school start times. See Table 5 for the comparison of data on early and later school start times (93 paired samples). **(A)** Simulations of the sequence of 10 sleep–wake cycles consisting of 2 last days of vacation (Sa and Su), 5 weekdays (Mo–Fr), two weekend days (Sa–Su), and the first weekday of the next week (Mo). **(B, C)** Two subintervals of this 10-day interval, for a weekday **(B)** and the last 3 days **(C)**.

Table 1 also lists the model parameters applied in the computations of the effects of three different weekday risetimes (wRTs) on the sleep–wake cycles (Figures 3–8; Tables 2–5). These computations (Table 2) show the effect of different amounts of advance shifts of wRT relative to the risetime (RT) on vacation (vRT = 9:00), either 8:00 or 7:00 or 6:00. For all these computations, bedtimes and risetimes on vacation were the same, i.e., vBT = 24:00 and vRT = 9:00, respectively (Figures 3A–C, respectively). The results given in Figures 3–6 indicate that irrespective of the shift of wRT, *ad lib* sleep on weekends always leads to the return of the risetime back to 9:00 (i.e., the same endogenously determined clock time as 1 week ago). Such rhythmostasis of *ad lib* sleep timing is achieved due to the circadian modulation *C(t)* (12) of the parameters of the sleep–wake-regulating process, *S(t)*, modeled as the alternations of wake (11a) and sleep (11b) phases of the cycle. In particular, the computations suggested that although sleep onset on Friday night is earlier after wRT = 6:00 than after wRT = 7:00 and that, in turn, sleep onset after wRT = 7:00 is earlier than after wRT = 6:00, the parameters of *ad lib* sleep at night between Friday and Saturday are modulated by the rhythmostat in such a way that this cycle ends up always at approximately 9:00, i.e., the same time as vRT (Figures 3–6; Tables 2 and 3).

**TABLE 2:** Sleep times computed for 10 sleep–wake cycles with three different weekday risetimes.

		Three wRTs, Figures 3–6											
		wRT at 6:00				wRT at 7:00				wRT at 8:00			
	Day	BT	RT	TiB	+W	BT	RT	TiB	+W	BT	RT	TiB	+W
Vacation	Sa	24.00	9.00	9.00	24.00	24.00	9.00	9.00	24.00	24.00	9.00	9.00	24.00
	Su	24.00	9.00	9.00	22.42	24.00	9.00	9.00	23.15	24.00	9.00	9.00	23.67
7	Mo	22.42	6.00	6.00	24.38	23.15	7.00	7.00	24.15	23.67	8.00	8.00	24.04
days of	Tu	22.80	6.00	7.58	23.92	23.30	7.00	7.85	23.98	23.71	8.00	8.33	24.00
the week	We	22.72	6.00	7.20	24.02	23.28	7.00	7.70	24.00	23.71	8.00	8.29	24.00
	Th	22.74	6.00	7.28	24.00	23.28	7.00	7.72	24.00	23.71	8.00	8.29	24.00
	Fr	22.74	6.00	7.26	25.22	23.28	7.00	7.72	24.69	23.71	8.00	8.29	24.28
	Sa	23.95	8.50	9.76	24.04	23.98	8.72	9.43	24.02	23.99	8.88	9.18	24.01
	Su	24.00	8.98	9.03	22.42	24.00	8.99	9.01	23.15	24.00	9.00	9.01	23.67
	Mo	22.42	6.00	6.00	24.38	23.15	7.00	7.00	24.15	23.67	8.00	8.00	24.04
Weekly averaged		23.05	6.78	7.73	24.00	23.47	7.53	8.06	24.00	23.78	8.27	8.48	24.00

BT, bedtime; RT, risetime; TiB, time in bed; +W: TiB + the following wake phase duration (i.e., the estimate of a period of the sleep–wake cycle). Weekly averaged: Weekly averaged sleep times (7 days, from Mo to Su/7). Clock time is given in decimal hours. The model parameters are given in Table 1, and the illustrations of computed and simulated sleep times (for each day of the week) are given in Figures 3–6.

**TABLE 3:** Simulation of sleep times and observed sleep times in the whole sample and its halves.

Simulation vs averaged data			Simulation			Whole dataset and its two halves					
			wRT at		Whole (Figure 6)			Earlier wRT		Later wRT	
Sleep time (abbreviation)			6:00	8:00	7:00	Mean	SD	Mean	SD	Mean	SD
Weekday	Bedtime	(wBT)	22.69	23.70	23.26	23.01	1.16	22.72	0.96	23.30^***^	1.26
	Risetime	(wRT)	6.00	8.00	7.00	6.97	0.65	6.47	0.36	7.48^***^	0.46
Weekend	Bedtime	(fBT)	23.98	24.00	23.99	23.96	1.25	23.75	1.10	24.18^***^	1.35
	Risetime	(fRT)	8.74	8.96	8.85	8.93	1.12	8.73	1.20	9.12^***^	1.00
Time in	Weekday	(wTiB)	7.31	8.30	7.74	7.96	1.10	7.75	0.99	8.18^***^	1.15
bed	Weekend	(fTiB)	8.76	8.94	8.86	8.96	1.00	8.99	0.93	8.94	1.07
Weekend–	Bedtime	(fwBT)	1.29	0.30	0.73	0.95	0.63	1.03	0.68	0.88^***^	0.57
weekday	Risetime	(fwRT)	2.74	0.94	1.85	1.95	1.12	2.26	1.18	1.64^***^	0.95
difference	Time in bed	(fwTiB)	1.45	0.64	1.12	1.00	0.78	1.24	0.82	0.76^***^	0.65

The shifts in *φ*_max_, *t2,* and *t1* were not proposed to be absent (0.0 h) for three preliminary simulations of sleep times (Table 1) and in the simulations of sleep times in the whole dataset (n = 1,048; Table 3); wBT and fBT, wRT and fRT, and and wTiB and fTiB: Weekday and weekend bedtime, risetime and time in bed, respectively; fwBT, fwRT, and fwTiB: Gaps between weekends and weekdays in these sleep times. Mean and SD: sleep time averaged over samples and its standard deviation; ^***^ *p* < 0.001 for *t* in Student’s t-test for equality of mean sleep times in two halves of samples (i.e., after the division of the whole dataset intro two halves with earlier and later wRT; n = 524 for each half). Clock time is given in decimal hours. See Figure 6 for the illustration of 10 simulated sleep cycles, Figure 3 for the illustration of two of these cycles, Figures 4, 5 for the illustrations of short subintervals (around bedtimes and risetimes), and Tables 1 and 2 for the model parameters and sleep times predicted for 10 days, respectively.

**TABLE 4:** Simulated sleep times and sleep times observed before and during lockdown.

Simulation vs averaged data			Before and during lockdown (Figure 7)					
			Simulation: wRT		Before		During	
Sleep time			7.0 + 0.0 h	8:0 + 0.2 h	Mean	SD	Mean	SD
Weekday	Bedtime	wBT	23.26	23.90	23.32	1.04	24.01^***^	1.10
	Risetime	wRT	7.00	8.20	6.95	0.73	8.08^***^	1.07
Weekend	Bedtime	fBT	23.99	24.20	24.03	0.97	24.43^***^	1.00
	Risetime	fRT	8.85	9.14	8.62	0.96	8.97^***^	1.02
Time in	Weekday	wTiB	7.74	8.30	7.63	0.86	8.07^***^	1.02
bed	Weekend	fTiB	8.86	8.94	8.59	0.89	8.54	0.89
Weekend–	Bedtime	fwBT	0.73	0.30	0.71	0.45	0.42^***^	0.43
weekday	Risetime	fwRT	1.85	0.94	1.67	0.87	0.89^***^	0.60
difference	Time in bed	fwTiB	1.12	0.64	0.96	0.59	0.47^***^	0.41

The shifts in φ_max_, *t2,* and *t1* (in h) were added to account for the delay in sleep times observed during lockdown, but these shifts were not required for the simulation of sleep times before lockdown.

*** *p* < 0.001 either for paired Student’s t-test or for the related-samples Wilcoxon signed-rank test of whether mean values obtained for 74 pairs of samples before and during lockdown are equal. See the parameters of the model (without shifts) in Table 1 and the illustration of 10 simulated sleep cycles in Figure 7.

**TABLE 5:** Simulated sleep times and sleep times observed during early and later school start times.

Simulation vs averaged data			Early and later school start time (Figure 8)					
			Simulation: wRT		Early		Later	
Sleep time			6.0 + 0.5 h	7.0 + 0.8 h	Mean	SD	Mean	SD
Weekday	Bedtime	wBT	23.19	24.06	23.05	1.24	23.59^***^	1.40
	Risetime	wRT	6.50	7.80	6.37	0.53	7.90^***^	1.03
Weekend	Bedtime	fBT	24.48	24.79	24.38	1.32	24.62^***^	1.44
	Risetime	fRT	9.24	9.65	9.58	1.09	9.77^***^	1.16
Time in	Weekday	wTiB	7.31	7.74	7.32	1.28	8.30^***^	1.04
bed	Weekend	fTiB	8.76	8.86	9.20	0.85	9.15	0.99
Weekend–	Bedtime	fwBT	1.29	0.73	1.33	0.59	1.03^***^	0.59
weekday	Risetime	fwRT	2.74	1.85	3.21	1.30	1.87^***^	0.87
difference	Time in bed	fwTiB	1.45	1.12	1.88	0.92	0.84^***^	0.73

The shifts in φ_max_, *t2,* and *t1* (in h) were added to account for the delays in sleep times observed in the student samples.

*** *p* < 0.001 either for paired Student’s t-test or for the related-samples Wilcoxon signed-rank test of whether mean values obtained for 93 pairs of samples with early and later school start times are equal. See the parameters of the model (without shifts) in Table 1 and the illustration of 10 simulated sleep cycles in Figure 8.

Since *S(t)* is modulated by the body clocks (*C(t)* > 0), the variation in all characteristics of the cycle, with the exception of wRT, reflects the permanent control of these clocks. The shifts in the phase of the cycle relative to the phase of the clocks are not predicted. Sleep onset on each of the weekdays and on each of the weekends occurs exactly at that moment when *S(t)* reaches its highest expected buildup, *S*_*d*_ *(t)*, set by the rhythmostat. In other words, *S(t)* does not cross *S*_*d*_ *(t)* on any day of the week. This implies that, during weekdays, there is no PoW, i.e., an extension of the wake (buildup) phase of the sleep–wake cycle, beyond the time determined by this endogenous regulator of the sleep–wake cycle (Figures 3–6; Tables 2 and 3). Consequently, EWU from Monday to Friday and *ad lib* wakeups on the following weekend cannot provoke two shifts, back and forth (on the weekend and weekdays, respectively) of the sleep–wake cycle relative to the unchanged phase of circadian clocks (the so-called “social jetlag” (Wittmann et al., 2006)). The result indicating that sleep onset occurs exactly at *S*_*d*_ *(t)* (i.e., a time point of the highest buildup of *S(t)* set by the rhythmostat) implies that the everyday control of the body clocks over the cycle cannot be lost. It remains in sync with these clocks throughout the week.

To sum up, the computations showed that due to the permanent circadian modulation, *C(t)*, of *S(t)*, a phase position of sleep onset at the end of each day of the week coincides with the highest buildup, *S*_*d*_ *(t)*, set by the rhythmostatic regulator of the sleep–wake cycle. Therefore, irrespective of how large the advance of wRT was, the night between Saturday and Sunday (i.e., the second night of *ad lib* sleep) ends by waking up at 9:00, and the following sleep onset on Sunday occurs exactly at 24:00 after *ad lib* wakefulness (Figures 3–8). Therefore, this rhythm-regulating mechanism does not reveal any “social jetlag,” i.e., the advancing and delaying shifts of the sleep–wake process *S(t)* relative to its circadian modulator, *C(t)*, during weekdays and weekends, respectively.

#### 2.1.3 The pivotal feature of the two-process model in light of computations

Any version of the two-process model postulates such a pivotal feature of the sleep–wake-regulating mechanism as an accumulation of “sleep debt” caused by PoW and its “paying off” during the following (recovery) sleep (Daan et al., 1984; Putilov, 1995). This prediction is illustrated by 1-h PoW in Figure 1. Moreover, one additional curve is shown in Figure 6A to illustrate such an accumulation of “sleep debt” during PoW beyond the highest buildup, *S*_*d*_ *(t)*, for a much longer time interval, including the whole nighttime and the following daytime. The curve starts at night between the last two vacation days (Saturday and Sunday) to build up during the 24-h interval of PoW. The model predicts that when such a buildup of *S(t)* above the highest expected buildup, *S*_*d*_ *(t)*, is terminated, the position of *S(t)* on the graph is profoundly higher than the position of *S*_*d*_ *(t)*. The following (recovery) sleep is expected to increase in duration. Such a recovery sleep is shown in Figures 1A, B. However, as mentioned previously, the rhythmotatic regulator prevents PoW (i.e., the buildup above *S*_*d*_ *(t)*) on any of five weekdays after EWU (Tables 2–5; Figures 2–8). On each of these days, the wake phase is terminated exactly at *S*_*d*_ *(t)*, i.e., the highest expected level of buildup of *S(t)*. Sleep is not initiated at above the *S*_*d*_ *(t)* level on any day of the week, i.e., not only after the reduced sleep episodes on weekdays but also after *ad lib* sleep episodes on the 2 days of the weekend (Tables 2–5; Figures 2–8). In addition, sleep on these 2 days is always terminated exactly at *S*_*b*_ *(t),* i.e., the lowest expected decay of *S(t)*. Therefore, wakefulness on any of the weekdays cannot be viewed as PoW, causing accumulation of “sleep debt.” If nothing is accumulated during weekdays, nothing is “paid back” during the following weekend. The computations suggested that the rhythmostat prevents sleep extension on weekends; i.e., it excludes oversleeping on weekends.

Consequently, the results of computations of the timing and duration of the wake and sleep phases of the cycle do not reveal any positive correlation between the duration of preceding wakefulness, i.e., the duration of the buildup of *S(t)*, and the duration of the following sleep, the duration of the decay of *S(t)*. Such a correlation can be expected in the case of accumulation and “paying back sleep debt.” The weekly averaged period of the sleep–wake cycle is always equal to 24 h, which implies that when wRT occurs earlier and the weekly averaged sleep duration is shorter, the weekly averaged duration of wakefulness is not also shorter. Instead, it is longer (Table 2). This relationship between durations of the preceding wake phase and the following sleep phases of the circadian sleep–wake cycle suggests a negative rather than positive correlation between these durations. Moreover, the computations predicted very similar durations of *ad lib* sleep on the night between Saturday and Sunday after either a large or a small advancing shift of wRT relative to vRT (Tables 2–5; Figures 3–8).

To sum up, the concepts of weekday accumulation of “sleep debt” and its weekend “paying off” (Roenneberg et al., 2012; Kitamura et al., 2016; Shen et al., 2021) and the concepts of “catch-up” (Kim et al., 2011) or “compensatory” sleep (Åkerstedt et al., 2019) cannot be supported by the results of present computations, suggesting that weekend time in bed after early wRT is not significantly longer than that after later wRT. Therefore, weekend *ad lib* sleep can be viewed as the sleep of normal, adequate, endogenously determined duration. Such sleep is not a kind of recovery sleep proposed by such concepts, as the concepts of catch-up and compensatory sleep postulate, explicitly or implicitly, that “sleep debt” was previously accumulated due to EWU on weekdays.

### 2.2 Four counterintuitive predictions of the *in silico* study

The results of calculations given in Figures 1–5 allowed highlighting their counterintuitive predictions. This list of such predictions includes four predictions: 1) the restoration of normal sleep duration and timing after any manipulation with durations of sleep and wake phases of the sleep–wake cycle is invariantly rapid; 2) sleep on weekends after weekday EWU without any PoW cannot be extended, and therefore, this sleep has normal, adequate duration, i.e., it is not the so-called “recovery sleep;” 3) since sleep on weekends has normal, adequate duration, any loss of sleep on weekdays due to earlier wakeups is an irrecoverable sleep loss; i.e., this loss cannot be compensated by an extension of *ad lib* weekend sleep; and 4) irrespective of the amount of such deadweight sleep loss on weekdays, the control of the circadian clocks over the sleep–wake cycle is not interrupted throughout any of the 7 days of the week; e.g., the cycle phase cannot be shifted relative to the unchanged circadian phase.

#### 2.2.1 The invariant return to normal (endogenously determined) sleep times

The computations suggested that just 1 day with *ad lib* sleep is sufficient for restoring the baseline (endogenously determined) sleep timing and duration after various manipulations with the duration of the wake and/or sleep phase of the sleep–wake cycle. In particular, the model-based calculations revealed that the duration of the restoring process shown in Figures 1–6 was very similar after a larger and a smaller reduction in the sleep phase for either 1 or 5 days. Thus, the restoring process was found to be, counterintuitively, short and practically identical after any of the most typical manipulations with the sleep and wake phases.

Furthermore, the model (Putilov, 1995) is a traditional model in representing the somnostatic (homeostatic) process, *S(t)*, as the alternations of an inverse exponential curve for the wake phase (11a) and an exponential curve for the sleep phase (11b) (Daan et al., 1984). However, it additionally postulates the modulation of parameters of *S(t)* by the body clocks represented by a 24-h sine-form function *C(t)* (12) (Putilov, 1995). At baseline, when sleep duration and timing are determined endogenously, *S(t)* oscillates between its highest and lowest buildup and decay, *S*_*d*_ *(t)* and *S*_*b*_ *(t)*, which might represent either sleep onset and offset (Figures 1, 2) or bedtimes and risetimes (Figures 3–8; Table 2). However, humans do not always obey the signal of falling asleep sent by their internal device. They might be forced (or eager to voluntarily) to delay the time to go to bed. Therefore, their wake phase might be extended due to such PoW. Similarly, they can reduce their sleep phase by EWU, i.e., as compared to the time of spontaneous waking-ups at *S*_*b*_ *(t)*. During such PoW, *S(t)* continues to build up after crossing *S*_*d*_ *(t)*, but, despite this, the following sleep episode is always terminated at endogenously determined *S*_*b*_ *(t)*. Due to EWU, *S(t)* does not reach *S*_*b*_ *(t)*, but, despite this, the following sleep episode is always initiated at *S*_*b*_ *(t)*.

Figure 1A illustrates the effects of PoW leading to a further increase in *S(t)* beyond the highest allowed buildup, i.e., further increase in *S(t)* after crossing *S*_*d*_ *(t)*. Such an additional increase in *S(t)* above *S*_*d*_ *(t)* can be interpreted as the accumulation of “sleep debt.” It is “paid off” during the following (recovery) sleep episode, which is expected to be longer than the normal sleep episode started at the same time point after reaching *S*_*d*_ *(t)*. Figure 1 shows the identity of the processes of restoration of the baseline duration and timing of sleep after 1 day with such PoW (Figure 1A), 5 days with PoW (Figure 1B), and 5 days of the combination of such PoW with EWU (Figure 1C). Superposition of two sleep–wake cycles in these graphs illustrates that due to the circadian modulation *C(t)* of the parameters of *S(t)*, it takes only one night of *ad lib* sleep after any of these three manipulations with the wake phase duration to reestablish the baseline times of sleep onset, offset, and duration (23:00, 7:00, and 8.00 h, respectively), i.e., a normal alternation of two phases of this process between *S*_*d*_ *(t)* and *S*_*b*_ *(t)* determined by the rhythmostat. Therefore, the duration of such reestablishment is not affected by the amount of the previous sleep loss. The examples are the number of days with PoW, either 1 day of such PoW (Figure 1A) or 5 (week)days of such EWU (Figure 1B), and a severe sleep loss due to the addition of EWU to PoW (Figure 1C).

Figure 2 illustrates that the effect of PoW on the rate of reestablishment of baseline times of sleep onset, offset, and duration (23:00, 7:00, and 8.00 h, respectively) is similar to the effect of EWU. Again, irrespective of the number of days with such manipulation, either 1 day (Figure 2A) or 5 (week)days (Figure 2B), only one night of *ad lib* sleep is required. The sleep phase is always initiated by the internal (rhythmostatic) device at *Sd(t)*, i.e., not only during baseline (unmanipulated) sleep but also during any of the days with EWU. Therefore, since the wake phase is not extended, none of EWU can lead to the accumulation of “sleep debt” on any of the days before and after it. Since there is nothing to be “paid off” during *ad lib* sleep after days with EWU without PoW, it would be more accurate to call such a sleep “relaxatory” rather than “recovery.” Thus, the computation results indicated that the duration of reestablishment of endogenously determined sleep times is not affected by the number of days of manipulation with duration of sleep or wake phase, either by means of EWU (Figures 2A, B) or PoW (Figures 1A, B) or by both means (Figure 1C).

Finally, Figure 3 shows similar results on the rate of the reestablishment of the baseline bedtime, risetime, and time in bed (24:00, 9:00, and 9.00 h, respectively) after three various earlier risetimes (ERTs). Irrespective of the size of the advancing shift of risetimes on each of the 5 (week)days, the restoration process requires only one night of *ad lib* sleep (Figures 3–5; Table 2). Three calculations were performed for different shifts of risetime: these was either a small shift (at just 1 h) or the most usual shift (at 2 h) or a large shift (at as many as 3 h) relative to the risetime on free days (e.g., the risetime at 9:00 h on vacation, vRT, when the cycle is unchallenged by any forced or voluntary shifts of bedtime and risetime). It was found that, after allowing one night with *ad lib* sleep, the risetime rapidly returns back to 9:00, i.e., the same time as at baseline (Table 2). Again, the circadian modulation, *C(t)* (12), of the parameters of the sleep–wake-regulating process *S(t)* (11) ensures such rhythmostasis, i.e., when *ad lib* sleep on the night between Friday and Saturday is sufficient to end up sleep at night between Saturday and Sunday always near 9:00.

Figures 3A, C illustrate that after ERT at 6:00 and 8:00 (2-h difference between two ERTs), the bedtime on Friday is set by the rhythmostat at approximately 22:45 and 23:45 h, respectively (1-h difference between them), and the risetimes on Saturday morning (i.e., after the first night of *ad lib* sleep) became almost the same (a difference became negligible). When sleep phases are initiated later, the process is “accelerated” to equalize risetimes on Saturday morning; i.e., the following sleep episodes are initiated after days with earlier and later wakeups (Table 2; Figures 3A, C). Again, any *ad lib* sleep episode after termination of EWU/ERT can be named “relaxatory” rather than “recovery” sleep due to the absence of PoW in the preceding weekdays. Thus, the rhythmostatic influence on the parameters of the sleep–wake-regulating process ensures the invariantly rapid return to the baseline risetime, bedtime, and time in bed on Sunday (approximately 9:00, 24:00, and 9.00 h, respectively) despite the difference in the advancing shift of wRT and the existence of the following, albeit less prominent, difference in bedtime prior to *ad lib* sleep.

Altogether, the modulating influence of the circadian clocks, *C(t)*, on the parameters of the somnostatic process, *S(t)*, explains a close similarity of any of the returns to the baseline sleep and wake phases of the 24-h cycle after either forced or voluntary manipulations with durations of these phases, e.g., after 1 day/5 days of larger/smaller reductions/extensions of the sleep/wake phase of the cycle. The circadian clocks govern the sleep–wake regulatory process in such a way (11,12) that, after any shifts in the times of going to bed and/or getting up (or bedtimes and/or risetimes), only one night of *ad lib* sleep is necessary for the reestablishment of the baseline times of sleep onset, sleep offset, and sleep duration (or the baseline bedtime, risetime, and time in bed) determined by this endogenous sleep–wake-regulating mechanism named “rhythmostat.”

#### 2.2.2 The irrecovery nature of sleep on weekends

As mentioned previously, such a pivotal feature of the sleep–wake-regulating mechanism as a response to PoW by accumulation of “sleep debt” with its “paying off” during the following (recovery) sleep is postulated by any version of the two-process model (Daan et al., 1984; Putilov, 1995). Such a model predicts that when a PoW-associated buildup of *S(t)* after crossing *S*_*d*_ *(t)* (i.e., the highest allowed buildup) is stopped, the position of *S(t)* on the graph (Figure 1) is always higher than that of *S*_*d*_ *(t)* at the same time point. Because it is expected that the following sleep is increased in length and intensity relative to the length and intensity of sleep started at *S*_*d*_ *(t)* at the same time point, such a sleep episode can be named “recovery sleep” (Figures 1A, B). However, EWU and ERT do not lead to any amount of PoW (i.e., sleep onset is set at 23:00 h or earlier in Figure 2, or risetime is set at 24:00 h or earlier in Figures 3–5 and Table 2). Therefore, no any accumulation of “sleep debt” is postulated during wakefulness after EWU/ERT, and, consequently, there is no “payment off” during *ad lib* sleep in the weekend nights following such EWU/ERT.

When people voluntarily get out of bed on Monday morning, they might want to go to sleep again at the same later time as they have chosen a day earlier, in the beginning of the night between Saturday and Sunday. However, their internal device forces them to go to sleep on weekdays somewhat earlier, thus preventing any PoW, a cause of accumulation of “sleep debt.” In other words, the rhythmostat usually does not allow PoW on any of the 5 weekdays after EWU/ERT by controlling the bedtime set exactly at *S*_*d*_ *(t)* (Figures 2–5). Therefore, the wake phase is always terminated exactly at this time point, *S*_*d*_ *(t)*, which is the maximal allowed level of buildup of *S(t)* both after a reduced sleep episode on any of the 5 weekdays and after any of *ad lib* sleep episodes on a 2-day weekend. Moreover, *ad lib* sleep on 2 days of the weekend is always stopped exactly at *S*_*b*_ *(t),* which is the minimal allowed decay of *S(t)* set by the same rhythmostatic device (Figures 2–5). As a result, wakefulness on weekdays after EWU/ERT cannot lead to PoW associated with the accumulation of “sleep debt.” Nothing can be accumulated during weekdays after EWU/ERT, and nothing can be “paid back” during the following 2 days of the weekend. Therefore, a sleep episode on any of these days after termination of weekday EWU/ERT can be named “relaxatory” rather than “recovery” sleep. Overall, the rhythmostat excludes oversleeping on any of the 7 days of the week, and, therefore, weekend *ad lib* sleep can be viewed as sleep of normal, adequate, endogenously determined duration rather than as a recovery sleep caused by “sleep debt” during the 5 previous weekdays.

#### 2.2.3 The irrecoverable loss of sleep on weekdays

It is natural to expect that weekend time in bed (or sleep duration) after a small shift in wRT would be significantly longer than that after a large shift. Despite this, time in bed (or sleep duration) on the weekend after 5 days of a small wRT shift is not predicted to exceed that after a large wRT shift (Figures 3–6; Table 2). Since, irrespective of the amount of deadweight sleep loss caused by early weekday wakeups, the duration of sleep on weekdays cannot be extended, the clock time for risetime after sleep on the night between Saturday and Sunday remains the same after either earlier or later weekday risetimes. In other words, the rhythmostat does not allow oversleeping on weekends, i.e., *ad lib* weekend sleep cannot be extended beyond its normal duration determined by this internal device; it has normal, adequate duration irrespective of the amount of sleep loss on the previous weekdays. Therefore, any EWU leads to irretrievable sleep loss. Notably, such a deadweight sleep loss is the only sleep disturbance caused by EWU/ERT. Overall, despite complete freedom to sleep in and nap during the weekend, the rhythmostat prevents oversleeping on weekends, but it is designed to restore normal, endogenously set sleep timing and duration after one night of *ad lib* sleep. Since sleep loss on weekdays after ERT/EWU is irrecoverable, such a deadweight sleep loss seems to be the only disturbing effect on the sleep–wake cycle caused by these EWU/ERT on 5 weekdays.

#### 2.2.4 The uninterrupted circadian control over the process of sleep–wake regulation

The rhythmostat model provides an explanation of the rapid and invariant return of sleep timing and duration back to their normal ranges after any forced or voluntary manipulation with the lengths of the sleep and wake phases of the sleep–wake cycle. Since *S(t)* is permanently modulated by the circadian clocks (*C(t)* > 0), sleep onset after each morning EWU/ERT as well as after each weekend risetime is set exactly at that moment when *S(t)* reaches its highest allowed buildup, *S*_*d*_ *(t)*. Therefore, the extension of the wake (buildup) phase of the sleep–wake cycle beyond the time determined by this endogenous regulator of the sleep–wake cycle (i.e., PoW) cannot be observed during weekdays (Figures 2–6; Table 2). *S(t)* can reach but cannot cross *S*_*d*_ *(t)* on any day of the week because, at the moment of crossing *S*_*d*_ *(t)*, the internal device initiates an all-night sleep episode. For example, the calculations of the effects of three ERTs suggested that due to the permanent circadian modulation *C(t)* of *S(t)*, a phase position of sleep onset at the very end of each day of the week coincides with the highest allowed buildup, *S*_*d*_ *(t)*, set by the rhythmostat.

Figures 4, 5 illustrate that, irrespective of how large an advance of wRT was relative to the baseline risetime and irrespective of how large an advance of weekday bedtime was relative to the baseline bedtime, each weekend ends by awakening at approximately 9:00 after *ad lib* sleep at night between Saturday and Sunday, and the following bedtime scheduled at night between Sunday and Monday after the previous *ad lib* wakefulness occurs exactly at 24:00 h (Table 2). In other words, when an advance of weekday risetime is large (e.g., ERT at 6:00), risetime and bedtime return to 9:00 and 24:00 h, respectively, at the end of the week due to the modulating influence *C(t)* on *S(t)*. If an advance is, instead, small (e.g., ERT at 8:00), risetimes and bedtimes also return to 9:00 and 24:00, respectively, at the end of the week due to such a modulating influence. Consequently, earlier wakeups from Monday to Friday and *ad lib* wakeups at the end of the following weekend nights cannot lead to any phase shifts in the sleep–wake cycle relative to the unchallenged phase of the circadian clocks. The occurrence of bedtime exactly at *S*_*d*_ *(t)*, i.e., at the time point of the highest buildup of *S(t)* set by the rhythmostat, implies that the influence of the body clocks on the sleep–wake cycle cannot be disrupted, and therefore, the cycle remains in sync with these clocks on any of the 7 days of the week.

The same is true not only for ERT/EWU but also for PoW (Figures 1A, B). Since *S(t)* is permanently modulated by the body clocks (*C(t)* > 0), risetime after each PoW occurs exactly at the moment when *S(t)* reaches its allowed decay, *S*_*b*_ *(t)*, set by the rhythmostat (Figure 1). Consequently, an extension of the wake phase due to PoW and *ad lib* wakefulness on the following day cannot cause the phase shifts in the sleep–wake cycle relative to the unchanged phase of the circadian clocks. The occurrence of risetime exactly at *S*_*b*_ *(t)*, i.e., the time point of the lowest decay of *S(t)* set by the rhythmostat, points at the uninterrupted control of the circadian clocks over the sleep–wake cycle throughout each day of the week. Due to such a permanent modulation of the parameters of *S(t)* by the body clocks (*C(t)* > 0), the sleep–wake cycle and these clocks do not lose synchrony both during and after PoW. Their synchrony can be disturbed neither by shifts in the times to go to bed at later hours nor shifts in the times to get up at earlier hours. Therefore, the delaying or advancing shifts of sleep onset and offset after PoW and EWU/ERT can be simply viewed as the extensions of one phase at the expense of the reductions of another phase. This implies that they cannot be interpreted as phase shifts of the sleep–wake-regulating process, *S(t)*, relative to the phase of its permanent circadian modulator, *C(t)*. Such shifts are prevented by the mechanism of the entrained circadian clocks controlling the parameters of *S(t)* throughout the week and after such forced or voluntary manipulations as PoW and EWU/ERT, ensuring the rapid and invariant return to the baseline (endogenously determined) times of sleep onset, offset, and duration (or bedtime, risetime, and time in bed).

Overall, humans can be forced to (or can voluntarily) extend the wake phase (Figures 1A, B), reduce the sleep phase (Figures 2–5), and extend the wake phase with the following reduction in the sleep phase (Figure 1C), but any such manipulations of the duration of cycle phases simply change the moment of switching between these phases without disturbing the permanent link of the circadian clocks with the sleep–wake process *S(t) via* their permanent modulating influence on the parameters of this process. During and after any manipulation of the duration of the sleep or wake phase, the process always remains under the control of the circadian clocks on any day of the week. Such a simple mechanism as the circadian modulation *C(t)* (12) of the parameters of the sleep–wake-regulating process *S(t)* (11) excludes any phase shifts in the sleep–wake cycle relative to the unchanged phase of the circadian clocks.

### 2.3 Results of the simulation study of sleep times from 1,048 samples

For the sake of simplicity and clarity of the present computations and simulations, sleep times (input of the model) were rounded off (Table 1). Despite such rounding, after averaging empirically evaluated sleep times from the whole dataset (n = 1,048), these mean sleep times were found to be in a good agreement with sleep times predicted by the computation of the effect of the 2-h advance of wRT relative to vRT (i.e., RT on the days on vacation when people are expected to sleep *ad lib* on any day of the week). This result is shown in the left part of Table 3 and Figure 6. Moreover, the computations predicted and the statistical analysis of empirical data confirmed that weekend times in bed in two halves of samples of the whole dataset with earlier and later wRT are almost identical despite shorter weekday time in bed in the former half compared to the latter half. This prediction implies that weekend “catch-up” or “compensated” sleep cannot exists because, in the case of the existence of such recovery sleep, weekend sleep is expected to be significantly longer after a shorter weekday sleep than after a longer weekday sleep. The analysis of empirical times in bed on weekends showed that as it is predicted by the present computations, the times in bed were not significantly different in two halves of the whole sample (Table 3). In contrast, these two halves were significantly different in all other sleep times reported in the right part of Table 3.

Sleep times predicted by the computation of the effect of the 2-h advance of wRT relative to vRT were also in good agreement with the subset of data on sleep times before lockdown (Table 4; Figure 7). As for the fitting sleep times after lockdown, they required a slight correction of the circadian phase. This phase is determined by the external light–dark cycle. It is expected that the 24-h pattern of elimination before and after lockdown was not the same. A later wRT during weekdays is expected to lead to a small delay in the light–dark cycle compared to the cycle during weekdays with earlier wRT in the weeks preceding lockdown. Therefore, significantly later risetimes and bedtimes on free days are expected for later wRT than those for earlier wRT. Indeed, the empirical results of the two halves of the whole sample with earlier and later wRT showed that they were additionally different in the 24-h pattern of exposure to light. It was found that weekend sleep times were slightly delayed after later wRT compared to those after the earlier wRT (Table 3). Since the sleep–wake cycle remains in synch with the circadian clocks entrained by the external light–dark cycle, such results suggested that the circadian phase in simulations must be set at a later time after later wRT to account for the difference in timing of light exposure after later and earlier wRT. Such an expectation was confirmed in simulations of sleep times after lockdown. These simulations suggested that RT and BT on weekends were significantly later during lockdown than before it (Table 4; Figure 7). Moreover, the simulations suggested that RT and BT on weekends were significantly later during later school start time than during early school start time (Table 5; Figure 8). In addition, these simulations accounted for a general delay in sleep timing in adolescents compared to people of younger and older ages.

Thus, in order to account for the differences in the circadian phase caused by the difference in the exposure to the light–dark cycle, the delays in the entrained sleep–wake cycles were additionally suggested for the simulations of sleep times during lockdown (0.2 h; Figure 7 and Table 4) and in adolescents, especially during earlier school start time (0.5 h and 0.8 h during earlier and later school start time, respectively, Figure 8 and Table 5). Notably, all such delaying shifts in the circadian timing due to changes in the pattern of light exposure were much smaller than those large (more than 1 h) shifts in the phase of the sleep–wake cycle relative to the circadian phase suggested by the concept of “social jetlag.”

As for the difference in wRT in the analyzed paired samples, the simulations shown in Figures 7, 8 take into account the difference between conditions in advance of wRT. This advance changed from 2 h before lockdown to only 1 h during lockdown (Table 4) and from 3 h during early school start time to only 2 h during later school start time (Table 5). Again, such changes in wRT resulted in the expected significant changes in weekday times in bed, but also as predicted by computations, the significant changes in weekend times in bed between two conditions were not detected by statistical analyses (Tables 4 and 5). Again, these conditions were significantly different in all other sleep times given in Tables 4 and 5.

To sum up, the simulations of empirical data on two subsets of the whole dataset and on two paired subsamples provided support for the model-based prediction that weekend time in bed is not significantly longer after a shorter previous weekday time in bed than after a longer previous weekday time in bed. This implies that weekend sleep cannot be viewed as a kind of recovery (i.e., “catch-up” or “compensatory”) sleep and that an earlier wRT simply leads to a larger deadweight loss of sleep on weekdays without any disturbance in the permanent control of the circadian clocks over the time course of the sleep–wake-regulating process, *S(t)*. This control is emphasized by the influence of the times of transitions between the wake and sleep phases of the sleep–wake cycle. As predicted, it occurred at *S*_*d*_ *(t)*. The permanent control over such a transition is ensured by the circadian modulation *C(t)* of the parameters of this process of sleep–wake regulation.

## 3 Discussion

The basic properties of biological time-measuring systems have easily lent themselves to mathematical modeling that is often applied together with the experimental approaches to predict findings of future studies and to provide a deeper insight into rhythmic phenomena in the living nature. However, mathematical modeling was not implicated into questionnaire studies of human sleep timing on weekdays and weekends to test several widely held concepts (Strughold, 1971; Wittmann et al., 2006; Kim et al., 2011; Roenneberg et al., 2012; Kitamura et al., 2016; Åkerstedt et al., 2019; Shen et al., 2021) explaining the reaction of mechanisms of sleep regulation to early wakeups on 5 weekdays and the following *ad lib* sleep during 2-day weekends. The present paper addresses the question of whether these concepts can be validated against the results of simulations of sleep times with the rhythmostatic version (Putilov, 1995) of the two-process model of sleep–wake regulation (Daan et al., 1984). The following questions were asked: can early weekday wakeups cause “social jetlag” that postulates the conflict between social and biological clocks that leads to the back and forth shifts of sleep–wake cycles relative to the circadian phase remaining unchanged on weekends and weekdays, respectively? Can “sleep debt” be accumulated during weekdays to be “paid off” during weekends? Or, in other terms, can people “catch up” (or “compensate”) on sleep on weekends? The present results of model-based computations and simulations suggested that the answer to such questions is no, they cannot. The counterintuitive predictions of the model-based simulations included the following: 1) only one night of *ad lib* sleep is sufficient to restore the endogenously determined sleep times after various manipulations of the duration of the sleep or wake phases of the sleep–wake cycle, such as 1 day/5 days of larger/smaller reduction/extension of the sleep/wake phase of the cycle; 2) sleep loss on weekdays is irrecoverable; 3) irrespective of the amount of this loss, sleep on weekends is not elongated; and 4) the control of the circadian clocks over the sleep–wake-regulating process is not disrupted during the week. It was demonstrated that the parameters of the sleep–wake-regulating process are modulated by the circadian clocks in such a way that this process remains in synch with these clocks throughout the week. Such a mechanism of the circadian control of the parameters of the sleep–wake process excludes any possibility of phase shifts in the sleep–wake cycle relative to the phase of the circadian clocks on any 7 days of the week. Moreover, it was shown that irrespective of the amount of deadweight sleep loss caused by early weekday wakeups, the duration of sleep on weekends cannot be prolonged beyond its normal length. Since the endogenous regulator prevents oversleeping on weekends, weekend *ad lib* sleep cannot be viewed as a kind of recovery sleep. It has an adequate, endogenously determined duration. Irrespective of the amount of deadweight loss of sleep on weekdays, the normal timing of sleep is restored after *ad lib* sleep at the end of each week, i.e., on the night between Saturday and Sunday. These results on simulations of sleep times deepen our understanding of the mechanisms underlying the responses of the sleep–wake cycle to early weekday wakeups.

### 3.1 Can early weekday wakeups cause “social jetlag”?

In particular, the simulations did not support the assumption that due to the conflict between social and internal (biological) timing, the sleep–wake cycle shifts back and forth relative to the unchanged phase of circadian clocks on weekends and weekdays, respectively (i.e., a variant of explanation of the gaps between weekday and weekend sleep times known as “social jetlag” (Wittmann et al., 2006)). Instead, these simulations confirmed by the results of analyses of datasets on weekday and weekend bedtimes and risetimes suggested that these shifts are not necessary to postulate for explaining the impact of early weekday wakeup on sleep timing and duration. Due to the permanent circadian modulation of the parameters of the sleep–wake-regulating process, this process does not shift back and forth throughout the week relative to the phase of the circadian clocks. Rather, this modulation allows the return of normal risetimes and bedtimes at the end of each week after smaller and larger shifts of wRT relative to *ad lib* risetime (e.g., vRT).

Can the phase of circadian clocks remain unchanged after earlier wRT compared to later wRT? It can, but only in the absence of differences between earlier and later wRT in the 24-h pattern of exposure to external light sources. It is reasonable to expect that earlier wakeups can slightly advance the timing of light exposure. Therefore, such wakeups can provoke a rather small advancing shift in the phase of the circadian clocks and, consequently, a similar in size and direction (i.e., also rather small) advancing shift in the phase of the sleep–wake cycle that remains under the control of the entrained circadian clocks. The results of analysis of the pairs of samples can exemplify this phase advance. For instance, a small but significant phase difference was found between sleep times collected during early and later school start times. Similarly, a delay in wakeups can provoke a relatively small delaying shift in the phase of the circadian clocks and, consequently, a similar in size and direction (i.e., also relatively small) delaying shift of the phase of the sleep–wake cycle. This phase delay was found in the analysis of the dataset of paired samples of sleep times before and during lockdown.

It is important to stress that all simulations of averaged sleep times suggested that such advancing and delaying shifts in the phase of the sleep–wake cycle were not as large (0.2 h–0.3 h) and cannot resemble more than a 1-h phase shift in this cycle postulated by the concept of social jetlag that suggests such a measure of the phase shift as a difference between the timing of sleep on weekdays and weekends (Wittmann et al., 2006).

Moreover, it is unlikely that such small shifts in body rhythms, including the sleep–wake cycle, after a small shift in the 24-h pattern of light exposure can cause the process resembling the process of adjustment of these rhythms to a much larger shift of the circadian clocks in response to a much larger shift in the external light–dark cycle, the phenomenon known under the original name “jetlag” (Cingi et al., 2018). After such a shift in the central circadian clocks, it takes time to shift all other circadian clocks, the clocks and circadian processes of lower hierarchical levels. Therefore, various diurnal cycles of the body functions, including the sleep–wake cycle, cannot rapidly reestablish their normal phase relationship with the central circadian clocks, and, hence, they cannot rapidly reestablish their link to the 24-h pattern of light exposure. After such much larger shifts of the central clock phase, it usually takes several days for these cycles to complete their adjustment, and this period is associated with adverse jetlag symptomology (Cingi et al., 2018). Given that after early weekday wakeups leading to a small shift in the phases of body rhythms, it need not initiate such a long process of reestablishment of phase relationships with the central circadian clocks, the adverse symptoms associated with such wakeups was not found to represent the travel-induced jetlag symptomatology (Tavares et al., 2020).

Overall, if, after a flight over several meridians, a drastically large shift in the circadian phase occurs in response to a similarly large shift of the external 24-h light–dark cycle (i.e., at least, several hours), the phases of the circadian clocks and sleep–wake-regulating process are only slightly and simultaneously shifted in response to a similarly slight change in the 24-h pattern of exposure to external light due to earlier weekday wakeups. Therefore, the permanent modulation of the parameters of this process by these clocks ensures the rapid, practically shift-independent return to the endogenously determined sleep duration and timing after *ad lib* sleep at the end of the week. Since the timing of light exposure is expected to be later after later weekday risetimes than after earlier weekday risetimes, the timing of weekend sleep is also expected to be later after later weekday risetimes.

To sum up, it is of importance to note that the shifts in the phase of the sleep–wake cycle detected in these analyzed samples with earlier and later wRT are small, and it is necessary to distinguish these shifts occurring under the permanent control of the circadian clocks from much larger shifts after transmeridian flights. They require a much longer process of adjustment of the sleep–wake cycle to the shifts of the phase of the central circadian clocks associated with jetlag symptomology. In contrast, early weekday wakeups disturb the sleep–wake cycle by reducing the duration of the weekday sleep phase without disturbing the phase relationship of the sleep–wake cycle with the circadian clocks. Since the phase of these clocks does no shift much due to a slight change in the external light–dart cycle, these clocks continue their modulation of the parameters of the process of sleep–wake regulation in such a way that, after *ad lib* sleep and wakefulness during 2 weekdays, both duration and timing of sleep are restored.

In addition, the results allow the prediction that the effect of change in sleep times caused by short interruptions of all-night sleep can be small and the return to normal sleep timing and duration can also require one night of *ad lib* sleep. If, for instance, an individual is forced to wake up in the middle of the night to spend in a wake state (and, of course, in darkness) the next 2 h before falling back to sleep, his/her next risetime and bedtime might be delayed and sleep duration might be shortened. However, due to the circadian modulation of the parameters of his/her process of sleep–wake regulation, it is likely that bedtimes and risetimes can return to their previous positions during the following *ad lib* sleep.

However, it remains to be further investigated whether such a rapid and invariant return to the endogenously determined sleep timing and duration can occur in response to the rotating shifts, e.g., on a day off after 3 days of 8-h work in the evening, then at night, and, finally, in the morning. Such large shifts of the intervals of wakefulness can lead to large shifts in the 24-h pattern of light exposure and, hence, to the complication of the process of returning to normal sleep times.

### 3.2 Can people “pay off sleep debt” or “catch up” or “compensate” sleep?

The simulations also did not support what might be the most popular variant of explanation of the gaps between weekday and weekend sleep times, known as the concept of weekday accumulation of “sleep debt paid off” on the weekend (Roenneberg et al., 2012; Kitamura et al., 2016; Shen et al., 2021) that, explicitly or implicitly, suggests the recovery nature of weekend recovery sleep named, therefore, “catch-up” (Kim et al., 2011) or “compensatory” sleep (Åkerstedt et al., 2019). In fact, the concept of “social jetlag” also includes the concept of “sleep debt” as an explanation of the gap between weekday and weekend sleep duration (Wittmann et al., 2006). It is reasonable to expect that data on weekend sleep can suggest that 1) after waking up at 6:00, sleep loss is significantly larger than after waking up at 7:00, and, in turn, 2) after waking up at 7:00, sleep loss is significantly larger than after waking up at 8:00. Therefore, if these popular explanations are correct, the predictions about weekend sleep can be made as follows: 1) a significantly longer sleep duration on weekend is expected after weekday wakeup at 6:00 than after waking up at 7:00, and, in turn, 2) a significantly longer sleep duration on the weekend is expected after weekday wakeup at 7:00 than after waking up at 8:00. However, the present simulations supported the results of previous simulations (Putilov and Verevkin, 2018; Putilov, 2022; Putilov et al., 2022; Putilov, 2023) by confirming a model-based prediction that people cannot sleep significantly longer on the weekend after waking up on weekdays at 6:00 rather than at 7:00 and after waking up at 7:00 rather than at 8:00. These results imply that people did not accumulate “sleep debt” during weekdays after any early wakeups, and therefore, they can “pay off” nothing on weekends.

Although the present computations and simulations do not predict that “sleep debt” is accumulated on weekdays to be “paid back” on weekends, such a phenomenon is not excluded in the case of voluntarily or forced prolongation of wakefulness beyond the time of habitual falling asleep. After an early wakeup on Monday morning, people might want to go to sleep again at the same time as they did a day earlier, i.e., on the night between Saturday and Sunday, but their internal device sends them to bed somewhat earlier, thus preventing any prolongation of wakefulness, leading to accumulation of “sleep debt.”

Strogatz et al. (1986) came to the conclusion that 1) “circadian regulation dominates homeostatic control of sleep length and prior wake length in humans”; 2) “the circadian system, not the prior sleep–wake history, is most important in governing the length of unrestricted wake and sleep in humans”; and 3) “homeostatic mechanisms serve mainly to regulate the amount of slow-wave sleep (Borbély, 1982) rather than the overall duration of sleep.” These conclusions are not surprising because they were obtained after reanalysis of the spontaneous timing of 359 sleep–wake cycles recorded from 15 internally desynchronized human subjects. Indeed, the simulations based on the rhythmostat model predict that the homeostatic effect on sleep duration cannot be found in these subjects because they did not voluntarily prolongate their wakefulness phase beyond the time of falling asleep set by their internal sleep–wake-regulating mechanisms. However, their third conclusion seems to be too strong and cannot be generalized under any conditions. The exclusion is a condition of voluntarily or forced prolongation of wakefulness. The mechanism of accumulation and “paying off sleep debt” really exists, but to initiate this accumulation, it is necessary to obey the “falling-asleep signal” sent by the rhythmostat and prolong the wake phase.

The previous (Putilov and Verevkin, 2018; Putilov, 2022; Putilov et al., 2022; Putilov, 2023) and present simulations suggested that because “sleep debt” is not accumulated during weekdays, sleep on weekends has normal, adequate duration determined by the rhythmostat. These results were more recently supported by Klerman et al. (2021), who reanalyzed data of their previous experiments on the opportunity of sleep extension (14–16 h per day) and concluded that people cannot consistently “oversleep in the same way that they can consistently overeat.”

To sum up, the computations, simulations, and analysis of sleep times reported for more than 1,000 samples suggested that despite complete freedom to sleep in and nap during 2 weekend days, the rhythmostat prevents oversleeping and always restores sleep timing after two nights of *ad lib* sleep. The only disturbing effect of early weekday wakeups is an irrecoverable loss of sleep. Irrespective of the amount of such deadweight sleep loss, weekend sleep cannot be extended beyond its normal, adequate, endogenously determined duration. In other words, any earlier weekday wakeups lead to an irretrievable reduction in the night sleep duration, and this sleep disturbance is not relevant to the phenomena known under terms such as “jetlag,” “social jetlag,” and “sleep debt.”

### 3.3 Practical implications

The results on simulations of sleep times can have several practical implications. One such implication is the development of a methodology for studies of health impacts of such wakeups. Due to irretrievable sleep loss caused by too early wakeups, many workers/students, despite not being involved in the night shift or work, can have insufficient sleep. It is well established that sleep insufficiency provokes various health problems, including obesity and diabetes, cold, cardiovascular and infection diseases, cancers (Cohen et al., 2009; Buxton and Marcelli, 2010; Morselli et al., 2010; Thompson et al., 2011; von Ruesten et al., 2012; Soucise et al., 2017), and even early mortality (Cappuccio et al., 2010; Grandner et al., 2010). Therefore, the aims of medical research on the conflicts between social and internal (biological) clocks include the evaluation of adverse health impacts of irrecoverable sleep loss caused by too early weekday wakeups.

However, the studies of health impacts of the conflicts between social and internal (biological) clocks in the frameworks of concepts such as “social jetlag,” weekday “sleep debt,” and weekend “catch-up” or “compensatory” sleep often ignore the importance of the inclusion in analysis dependent variables such as weekday sleep duration (or weekday time in bed). It is calculated from the data on sleep onset and offset (or from data on bedtimes and risetimes). The analysis of data in these frameworks is focused on the measures of gaps between weekdays and weekends in sleep timing and duration. These gaps are also calculated from the same sleep onset and offset (or from bedtimes and risetimes). Therefore, both these gaps and weekday sleep duration (or weekday time in bed) are calculated from the same data on sleep onset and offset (or bedtimes and risetimes). The gaps correlate with weekday sleep duration (or weekday time in bed), but since it is difficult to account for this correlation in a single regression analysis aimed at predicting health variables, weekday sleep duration is simply excluded from such analysis. We previously confirmed the significance of the association of a reduced weekday time in bed with poorer health of university students and additionally showed that the association of health with late sleep timing and the gaps between weekdays and weekends in sleep timing and duration can be fully explained by the correlation of the reduction of time in bed on weekdays with later sleep timing and larger gaps (Putilov et al., 2023). This is not a surprise in light of present simulations that, on one hand, pointed at the existence of deadweight sleep loss on weekdays, but, on the other hand, questioned the recovery nature of *ad lib* weekend sleep and the phase-shifting nature of the gaps between weekends and weekdays in sleep times.

Moreover, in light of the results providing a deeper insight into the responses of sleep–wake regulation mechanisms to early weekday wakeups, the interpretation of previously reported findings on aversive health effects of such wakeups can be challenged. For instance, there exists an approximately 1-h difference between weekday risetimes of employed people in US counties located in close proximity to one another on two opposing sides—right and left—of the border between two time zones. This 1-h difference was found to be associated with a decrease in the health index by 0.3 standard deviations in people living in US counties on the late sunset side of a time zone border (i.e., on the right from this border) compared to those living in neighboring counties on the opposite (i.e., left) side of the border (Giuntella and Mazzonna, 2019). Since it was also found that these people slept, on average, 19 minutes lesser (Giuntella and Mazzonna, 2019), such sleep reduction might be a cause of their poorer health. Instead, the authors of this study explained their findings as “causal effects of social jetlag on health.” They, however, more generally defined “social jetlag” in this publication as “the discrepancy arising between biological and social times” (Roenneberg et al., 2012), rather than as a disturbance in the circadian organization of body functions resembling the travel-induced jetlag. The present simulations provided an explanation of the response of the rhythmostatic regulator to this conflict as a deadweight loss of sleep on weekdays rather than a disturbance in the circadian organization of body functions.

However, this explanation does not question the existence of such a conflict between social and internal (biological) clocks and its primary role in provoking weekday sleep loss. It seems that weekday sleep insufficiency as a health problem is mostly caused by the features of these clocks. They cannot be entrained to the social clocks that are not relevant to the 24-h cycle of light exposure (i.e., signals of an alarm watch). Instead, the internal clocks are entrained to this cycle and simply ignore the time signals from such social clocks. Therefore, the phases of body functions, including the positions of phases of sleep and wakefulness, remain to be set by the 24-h cycle of light exposure, while early weekday wakeups disturb sleep, sometimes making it insufficient rather than disturb the phase relationship of the sleep–wake cycle with the phase of circadian clocks.

Another example of practical implications of the simulation results is to answer to a question of how weekday sleep deficiency can be reduced in late individuals, e.g., with a late chronotype and in late adolescents and young adults. They are forced to live side by side with other individuals in our work/study cultural environment that remains to be biased toward the circadian clocks of early individuals. The simulations of sleep times in distinct chronotypes (Putilov and Donskaya, 2022; Putilov et al., 2022) suggested that irrespective of the chronotype, the reduction in weekday sleep cannot be compensated by the extension of weekend sleep beyond its normal, adequate, endogenously determined duration. The results of simulations of sleep times of age-matched early and late types reported by Putilov and Donskaya (2022) showed that they have identical homeostatic components of the sleep–wake regulation, while the only difference between them is the difference in the phase of circadian modulation. This difference determines their differences in sleep timing and duration on weekdays and weekends. This implies that the deadweight losses of sleep on weekdays are larger in late individuals than in early individuals under the conditions when they are forced to have similar risetimes on weekdays. Since the rhythmostat prevents oversleeping, any attempts of these late individuals to extend their weekend sleep are useless and cannot reverse the negative health effects of skimping on sleep during the week due to too early wakeups. Therefore, the model-based simulations can be recommended for calculating how large and dangerous their unrecoverable loss of sleep on weekdays is (Putilov et al., 2022). Consequently, the results of such calculations allow the recommendation of how large a delay of their weekday wakeups can be for equalizing them with early individuals on the amount of such loss (Putilov et al., 2022).

A counterintuitive empirical result was obtained by comparing weekday sleep losses in the groups of study participants with distinct chronotypes. It suggested a longer weekday time in bed in evening than morning types in adulthood (Putilov et al., 2020). Therefore, such recommendations are mostly necessary to develop for late adolescents who are forced to attend school early in the morning irrespective of their chronotype and age-specific tendency for lateness. A promising result of the application of such recommendations was reported by Zerbini et al. (2020), who demonstrated a possibility of reducing the gaps between weekdays and weekends in sleep timing and duration by controlling the light exposure at home. The authors concluded that this control might be effective in advancing melatonin secretion and sleep, thereby helping late chronotypes to better cope with early social schedules (Zerbini et al., 2020).

### 3.4 Further testing of the counterintuitive predictions of the model

Some findings of experimental studies provided support for predictions of the rhythmostat model. For instance, in 1995, the modulation of the time course of slow-wave activity (SWA) by the circadian clocks with a peak in the afternoon hours was predicted by the simulations based on the rhythmostat model (Putilov, 1995) (Table 1), while experimental results supporting this prediction were obtained only 20 years later (Lazar et al., 2015).

In this and previous publications (Putilov and Verevkin, 2018; Putilov, 2022; Putilov, 2023), the empirical data for simulations were collected from the literature to test the prediction of practically similar durations of *ad lib* weekend sleep after either a large or a small advancing shift in the weekday risetime, resulting in either a shorter or a longer weekday sleep duration, respectively. Analysis of sleep times in samples supported the prediction that, on weekends, time in bed is not significantly different after a shorter and a longer time in bed on weekdays caused by an earlier and later weekday risetimes, respectively. However, a field or laboratory experimental study has not been initiated so far for supporting this prediction. Such a study might, in particular, address the question of whether its results can provide further empirical evidence for the prediction of short and invariant duration of the reestablishment of baseline sleep duration after larger/smaller reduction/extension in the sleep/wake phase of the sleep–wake cycle.

For instance, such a study might be designed to collect data allowing the comparison of durations of *ad lib* sleep on a night between Saturday and Sunday before and after two different manipulations with weekday wakeups, e.g., 5 days of waking up earlier either 1 or 3 h earlier than in the previous night between Saturday and Sunday. The durations obtained after earlier and later wakeups are predicted to return to the same baseline (endogenously determined) durations after *ad lib* sleep at night between Friday and Saturday, and the durations of sleep at night between Saturday and Sunday before and after two such different manipulations are predicted to be practically identical (i.e., none of the significant differences between these two durations of sleep is expected to be revealed).

A similar design might be suggested for an experimental study aimed at comparing whether sleep durations between Saturday and Sunday are practically identical before and after two different manipulations with times to go to bed, e.g., prior to and following 5 days of prolongation of wakefulness for either 1 or 3 h.

Notably, a close similarity in the durations of sleep at night between Saturday and Sunday is expected to be confirmed in such an experimental study aimed at the confirmation of the counterintuitive model predictions, but the clock times of sleep on this night are not expected to show a close similarity. Instead, these clock times are expected to be slightly but significantly dissimilar due to an inevitable albeit small difference in the 24-h pattern of light exposure between the weeks with earlier and later weekday wakeups or the weeks with larger and smaller prolongation of wakefulness.

Recent experimental evidence that can be regarded as indirectly supporting the association of health problems with weekday sleep loss and non-recovery nature of weekday sleep is scarce. One of the examples is the study reported by Depner et al. (Depner et al., 2019; Depner et al., 2021), who demonstrated that sleep on weekends did not help in reversing a health problem associated with weekday sleep insufficiency. The authors concluded that 1) *ad lib* weekend sleep failed to prevent metabolic dysregulation during a repeated pattern of insufficient sleep and weekend recovery sleep (Depner et al., 2021) and 2) the effects of insufficient sleep, with or without weekend *ad lib* sleep, on a 24-h pattern of energy balance were not dissimilar (Depner et al., 2019). Another example is the study by Reichenberger et al. (2023), who found an increase in the heart rate and systolic blood pressure following successive nights of sleep restriction, and neither the heart rate nor systolic blood pressure recovered to baseline levels following two nights of *ad lib* sleep.

## 4 Materials and methods

The idea of “somnostat” was mentioned for the first time in the publication of a quantitative version of the two-process model (Daan et al., 1984). It was further developed into the rhythmostat model (Putilov, 1995) that considers a model of the thermostat of the relay type (Magnus, 1976) as a technical counterpart of the original “somnostat” model, i.e., the homeostatic process of sleep regulation or, in other terms, the process S (Daan et al., 1984).

### 4.1 Mathematical model of the thermostatic regulation of temperature

A detailed description of the relay thermostat model was provided by Magnus (1976). If *X(t)* is the current temperature, the equation for *X* can be obtained by considering the energy balance in a thermostatic system:

$$Ṡ={Ṡ}_{D}+{Ṡ}_{A},$$ (1)

where *Ṡ* is the production of energy by a heating device and *Ṡ*_*D*_ and *Ṡ*_*A*_ are the dissipated and accumulated energy, respectively.

When the heater is switched either on or off, *Ṡ* remains constant. Therefore, the dissipation of heat is proportional to the temperature:

$${Ṡ}_{D}=k*X.$$ (2)

If thermal capacity *m* is also a constant, the accumulation of energy is proportional to the thermal capacity:

$${Ṡ}_{A}=m*\dot{X}.$$ (3)

After substituting (2) and (3) in (1), Ṡ can be determined as follows:

$$Ṡ=m*\overset{\cdot }{X}+k*X,$$ (4)

and the time constant and the limits for temperature can be defined as

Ṡk=XuXl,

where $X_{u}$ and $X_{l}$ are the maximal and minimal temperatures, respectively. After substituting them into Eq. 4, the following equation is obtained:

$$T*˙X+X=\left\{\begin{array}{c} X_{u}\text{ for buildup phase},i.e.,\text{when the heater is switched on}\\ X_{l}\text{ for decay phase},i.e.,\text{when the heater is switched off} \end{array}\right..$$ (5)

For the initial *t* = 0, X = $X_{b}$ , and X = $X_{d}$ , Eq. 5 has the following solutions:

X=Xu‐Xu‐Xb*e−tT for buildup phase, (6a)

X=Xl‐Xd‐Xl*e−tT for decay phase. (6b)

If X is a temperature setpoint, the current temperature, $X\left(t\right)$ , oscillates up and down around this setpoint because it is impossible to fully exclude a delay between the result of temperature measurement and the following switching response. For instance, it takes time TT for an analyzer of temperature for reaching a signal from a switcher to the relay. Moreover, it is likely that a zone of insensitivity also exists, and within this zone, the regulator does not react to the deviation of $X\left(t\right)$ from X _S_. This phenomenon is known as hysteresis, and it has been mentioned in the note about “somnostat” by Daan et al. (1984) to stress the similarity between a thermostat and the hypothetical “somnostat.” Due to this hysteresis, the phase transitions (i.e., turning the heater on or off) occur at X = $X_{b}$ and X = $X_{d}$ rather than at X = X _S_.

X can be calculated for five time points. These are the beginning of temperature buildup, *t*_*b*_, the moment when temperature reaches the setpoint after the beginning of buildup, *t*_*bs*_, the beginning of temperature decay, *t*_*d*_, the moment when temperature reaches the setpoint after the beginning of decay, *t*_*ds*_, and the moment when the cycle repeats, *t*_*b+Ƭ*_.

$$X\left(t_{bs}\right)=X_{s},X\left(t_{d}\right)=X\left(t_{bs}+TT\right)=X_{d}\text{for buildup phase},$$ (7a)

$$X\left(t_{ds}\right)=X_{s},X\left(t_{b+\tau }\right)=X\left(t_{ds}+TT\right)=X_{b}\text{for decay phase}.$$ (7b)

After including these equations in (6) and excluding *t*_*bs*_ and *t*_*ds*_, the following equations are obtained:

Xd=Xu‐Xu‐Xs*e−TTT, (8a)

Xb=Xl‐Xs‐Xl*e−TTT, (8b)

G= 12 Xd− Xb=12Xu ‐ Xl*1 –e−TTT, (9)

where *G* is an amplitude of thermostatic oscillations.

The following are the limits for Eq. 9:

When *TT* → 0, *G* → 0, and when *TT* → ∞, *G* → $\frac{1}{2}\left(X_{u}-X_{l}\right)$ .

### 4.2 Mathematical model of the somnostatic regulation of sleep and wake states

In order to turn from this thermostat model (Magnus, 1976) to the model of “somnostat” (i.e., the process S proposed by Daan et al. (1984)), it is necessary to suggest that T (time constant) differs for the buildup and decay of two phases of oscillation of $X\left(t\right)$ around the setpoint:

$$T_{b}=Waking\;time\;/\;\ln\;\left[\left(X_{u}-X_{b}\right)/\left(X_{u}-X_{d}\right)\right],$$ (10a)

$$T_{d}=\left(24–Waking\;time\right)\;/\;\ln\;\left[\left(X_{d}-X_{l}\right)/\left(X_{b}-X_{l}\right)\right],$$ (10b)

where $T_{b}$ and $T_{d}$ are the time constants for the phases of buildup and decay, respectively (i.e., in the process S model, these are the wake and sleep phases, respectively).

### 4.3 Mathematical model of the rhythmostatic regulation of the sleep–wake cycle

The rhythmostatic version of the two-process model (Putilov, 1995) additionally postulates that the setpoint and time constants of such a homeostatic process, $X\left(t\right)$ , are modulated by the circadian clocks. In computations and simulations, this modulating influence of the body clocks can be introduced as the simplest (sine) periodic function with a circadian period. For instance, if *t1* and *t2* are the initial times for the buildup and decay phases, respectively (e.g., risetime and bedtime on vacation, vRT and vBT, respectively, in Table 1), this sleep–wake-regulating process, $X\left(t\right)$ , can be computed using the following equations:

$$X\left(t\right)=\left[X_{u}+C\left(t\right)\right]-\left\{\left[X_{u}+C\left(t\right)\right]-X_{b}\right\}*e^{-\frac{t-t1}{\left[Tb–\;k*C\left(t\right)\right]}},$$ (11a)

$$X\left(t\right)=\left[X_{l}+C\left(t\right)\right]-\left\{X_{d}-\left[X_{l}+C\left(t\right)\right]\right\}*e^{-\frac{t-t2}{\left[Td–\;k*C\left(t\right)\right]}},$$ (11b)

where

$$C\left(t\right)=A*\sin\left(2\pi *\frac{t}{\tau }+\varphi_{0}\right)$$ (12)

is a sine function with the circadian period τ representing the modulating influence of the circadian clocks (Putilov, 1995). In the present computations and simulations, this period was assigned to 24 h because the circadian clocks were proposed to always remain under control of (i.e., are entrained to) the external light–dark cycle with 24-h period.

This -rhythmostatic version (11, 12) (Putilov, 1995) of the two-process model of sleep–wake regulation (Daan et al., 1984) was applied for all present computations and simulations (Tables 1–3; Figures 1–8). The parameters of this model named “initial” (Table 1) were derived from data on the durations of recovery sleep after six gradually increasing intervals of extended wakefulness (Åkerstedt and Gillberg, 1981) and from data on the levels of SWA calculated for 10 naps (Dijk et al., 1987) and two recovery sleep episodes (Dijk et al., 1990; Dijk et al., 1991) (see the work of Putilov (1995) for more details). The simulation of such experimental data provided a possibility to use the measurements of relative SWA (rSWA) from the literature (Dijk et al., 1987; Dijk et al., 1990; Dijk et al., 1991) for calculations of $X\left(t\right)$ (11,12) (Putilov, 1995). To stress that the sleep–wake-regulating process (11, 12) in the present study resembles the process S proposed by Daan et al. (1984), $X\left(t\right)$ was renamed on “*S(t)*” in the description of computation and simulation results. The initial model parameters were applied for illustrating how the rhythmostat responds to PoW and EWU/ERT (Figures 1–5).

### 4.4 Collecting sleep times from the literature and their model-based simulations

The datasets with sleep times on weekdays and weekends were collected from the literature and included in Supplementary Materials as the three-page file in excel format. Details on this process of data collection were provided in previous publications (Putilov, 2022; Putilov, 2023). After submitting and publication of these two papers, the datasets were enlarged mostly by adding data from the current publication. Therefore, sleep times were statistically re-analyzed (Tables 3–5). In particular, the whole dataset was enlarged from 810 to 1,048 samples (page 1), the subset of sleep times collected before and during lockdown was enlarged from 31 to 74 paired samples (page 2), and the subset of sleep times obtained during early and later school start times was enlarged from 35 to 93 paired samples (page 3).

Statistical analysis was performed using the Statistical Package for Social Sciences (SPSS_23_, IBM, Armonk, NY, United States). The whole dataset (n = 1,048) was subdivided into two halves with earlier and later wRT (n = 524 and 524, respectively). Sleep times calculated for the two halves were compared using the independent-samples *t*-test (Table 3, right). A paired *t*-test was applied for comparing paired samples collected before and during lockdown and during early and later school start times (Table 4 and 5, respectively). The normal distribution was confirmed by the Kolmogorov–Smirnov test, and, if not confirmed, the related-samples Wilcoxon signed-rank test was additionally applied for comparing paired samples.

To perform the remaining computations and simulations (Figures 3–8), the initial parameters of the model were slightly modified to account for the difference in the time in bed in the datasets of the present study (approximately 9 h) from the experimental sleep durations obtained from the experimental examination of the effects of extension/reduction of wakefulness (Åkerstedt and Gillberg, 1981) (approximately 8 h) that were used by Putilov (1995) for the derivation of initial parameters (Table 1, three right and three left columns, respectively).

Moreover, for the sake of simplicity and clarity of present computations and simulations, sleep times were rounded off. For instance, vBT, vRT, and, consequently, vTiB were set at 24:00, 9:00, and 9.00 h, respectively. The three wRT were set at 6:00, 7:00, and 8:00, which provided a 3.00-h, 2.00-h, and 1.00-h advance, respectively, relative to the hypothesized preceding vRT.

In the simulations of the sleep times averaged over the whole set of samples (Table 3; Figure 6) and before lockdown (Figure 7; Table 4), these vocational sleep times (vBT and vRT) remained unshifted in the phase (0.00 h; Table 1). To simulate sleep times collected during lockdown and in adolescents during either early or later school start time (Figures 7, 8, respectively), the influence of changes in the 24-h pattern of exposure of the body clocks to external light sources were accounted for. The preliminary clock hours (24:00 and 9:00) were slightly corrected to obtain a better fit. These shifts of the circadian timing during lockdown and in adolescents attending school either early or later did not exceed 1 h (0.2 h and either 0.5 h or 0.8 h in Tables 4 and 5, respectively).

## 5 Conclusion

Here, mathematical modeling and model-based simulations were used to validate several widely held concepts proposed for explaining the responses of the mechanisms regulating the sleep–wake cycle to early wakeups on 5 weekdays, followed by *ad lib* sleep during 2-day weekends. In particular, the model-predicted sleep times were compared with sleep times reported in the literature for the purpose of answering the following questions: Can people experience “social jetlag” due to early weekday wakeups? Can people accumulate “sleep debt” during weekdays that is “paid off sleep debt” during weekends? Or, in other terms, can people “catch up” or “compensate” sleep during weekends? The answers to all such questions were no, they cannot. None of the results supported the assumption that, on weekends and weekdays, the phase of sleep–wake cycles can be shifted back and forth, relative to the unchanged phase of circadian clocks (“social jetlag”). Moreover, none of the results provided evidence for accumulation and “paying off sleep debt” during weekday wakefulness and the following weekend recovery (“catch-up” or “compensatory”) sleep, respectively. The results demonstrated that irrespective of the amount of deadweight sleep loss after early weekday wakeups, the sleep–wake cycle is permanently controlled by the circadian clocks throughout the week, thus remaining in sync with these clocks on weekdays and weekends, and this control does not allow oversleeping on weekends (i.e., an extension of *ad lib* sleep beyond its normal, adequate, endogenously determined duration). This implies, more or less, that early weekday wakeups are caused by the conflict between social and biological clocks. This conflict always leads to irretrievable sleep losses, and these losses can be viewed as the only health-damaging sleep disturbance caused by this conflict. It is necessary to directly test in an experimental study the counterintuitive predictions of model-based simulations of response of the internal sleep-regulating mechanism to the voluntary or forced manipulations of times to go to bed and waking up. For instance, such future experiment can be aimed at supporting the counterintuitive prediction of inability to extend sleep on weekends. Furthermore, counterintuitively, the simulations predicted that sleep loss on weekdays appears to be irrecoverable, it takes only one night of *ad lib* sleep for restoring normal sleep duration and timing after a larger and a smaller irrecoverable sleep loss on weekdays and after just 1 day and 5 days of irrecoverable sleep loss on weekdays, and irrespective of the amount of irrecoverable sleep loss on weekdays, the circadian clocks do not lose control over the sleep–wake cycle throughout the week.

## Data Availability

The original contributions presented in the study are included in the article/Supplementary Material; further inquiries can be directed to the corresponding author.
